# OTULIN protects the intestinal epithelium from apoptosis during inflammation and infection

**DOI:** 10.1038/s41419-023-06058-7

**Published:** 2023-08-19

**Authors:** Lien Verboom, Christopher J. Anderson, Maude Jans, Ioanna Petta, Gillian Blancke, Arne Martens, Mozes Sze, Tino Hochepied, Kodi S. Ravichandran, Lars Vereecke, Geert van Loo

**Affiliations:** 1 VIB Center for Inflammation Research, 9052 Ghent, Belgium; 2 Department of Biomedical Molecular Biology, Ghent University, 9052 Ghent, Belgium; 3 Centre for Inflammation Research, Institute for Regeneration and Repair, University of Edinburgh, Edinburgh, UK; 4 Department of Internal Medicine and Pediatrics, Ghent University, Ghent, Belgium

**Keywords:** Cell death and immune response, Chronic inflammation, Mucosal immunology

## Abstract

The intestinal epithelium is a single cell layer that is constantly renewed and acts as a physical barrier that separates intestinal microbiota from underlying tissues. In inflammatory bowel disease (IBD) in humans, as well as in experimental mouse models of IBD, this barrier is impaired, causing microbial infiltration and inflammation. Deficiency in OTU deubiquitinase with linear linkage specificity (OTULIN) causes OTULIN-related autoinflammatory syndrome (ORAS), a severe inflammatory pathology affecting multiple organs including the intestine. We show that mice with intestinal epithelial cell (IEC)-specific OTULIN deficiency exhibit increased susceptibility to experimental colitis and are highly sensitive to TNF toxicity, due to excessive apoptosis of OTULIN deficient IECs. OTULIN deficiency also increases intestinal pathology in mice genetically engineered to secrete excess TNF, confirming that chronic exposure to TNF promotes epithelial cell death and inflammation in OTULIN deficient mice. Mechanistically we demonstrate that upon TNF stimulation, OTULIN deficiency impairs TNF receptor complex I formation and LUBAC recruitment, and promotes the formation of the cytosolic complex II inducing epithelial cell death. Finally, we show that OTULIN deficiency in IECs increases susceptibility to *Salmonella* infection, further confirming the importance of OTULIN for intestinal barrier integrity. Together, these results identify OTULIN as a major anti-apoptotic protein in the intestinal epithelium and provide mechanistic insights into how OTULIN deficiency drives gastrointestinal inflammation in ORAS patients.

## Introduction

The intestinal epithelium represents the largest surface area of the body in contact with the external environment. The epithelium is renewed every 4–5 days through a balanced process of stem cell proliferation and transit-amplifying cell proliferation in the intestinal crypts and, conversely, detachment and cell death of terminally differentiated enterocytes at the villus apex [1]. The single-cell layer of intestinal epithelial cells (IECs) and overlying mucus layer forms a physical barrier that protects the body from invasion by gut microbes. Excessive death of IECs impairs the integrity of this barrier and compromises intestinal homeostasis, as is shown in inflammatory bowel disease (IBD) and in several mouse models of intestinal pathology [2].

Ubiquitination is a post-translational modification that regulates signaling pathways including innate immune signaling initiated by cytokine receptors such as tumor necrosis factor receptor 1 (TNFR1) and pattern recognition receptors (PRRs). Linear (M1) ubiquitination, controlled by the linear ubiquitin chain assembly complex (LUBAC) consisting of HOIL1 (Haem-oxidized IRP2 ubiquitin ligase 1), HOIP (HOIL1-interacting protein) and SHARPIN (Shank associated RH-domain interacting protein), promotes NF-κB activation but also prevents cell death induced by death receptors [3, 4]. M1 ubiquitin chains are removed by the deubiquitinating (DUB) enzymes OTULIN and CYLD [5–8]. OTULIN reduces cytosolic levels of M1 ubiquitin generated by LUBAC [9], but also promotes LUBAC activity by preventing its autoubiquitination [10, 11]. Patients with a homozygous loss-of-function mutation in *OTULIN* develop OTULIN-related autoinflammatory syndrome (ORAS), a potentially fatal autoinflammatory condition which can be managed by treatment with anti-TNF neutralizing antibodies [9, 10, 12]. Mice with loss-of-function mutations in *Otulin* are not viable and die midgestation [6, 7], but tissue-specific targeting studies confirmed the importance of OTULIN in the control of NF-κB and cell death responses [10, 13–15].

Tight regulation of cell death and NF-κB responses in the intestinal epithelium is important for intestinal homeostasis and pathology, as shown in many genetic mouse models [16–24] but also in patients with IBD [19, 25–27]. Since OTULIN is known to regulate NF-κB signaling and cell death responses [7, 11], and since clinical manifestations in ORAS patients include intestinal abnormalities [9, 10, 12, 28], we here investigated the role of OTULIN in the intestinal epithelium. For this, we generated mice that specifically lack OTULIN in IECs. These mice do not show a spontaneous phenotype but are more susceptible to experimental colitis induced by dextran sodium sulfate (DSS), and to intestinal infection with nontyphoidal *Salmonella*. These mice are also hypersensitive to a normally sublethal dose of TNF, which causes massive apoptosis of IECs inducing lethality. Consistent with this, OTULIN deficiency in IECs promotes the development of intestinal pathology in a chronic TNF-driven model of ileitis.

## Results

### OTULIN^IEC-KO^ mice are healthy and do not develop intestinal pathology

**Fig. 1:**
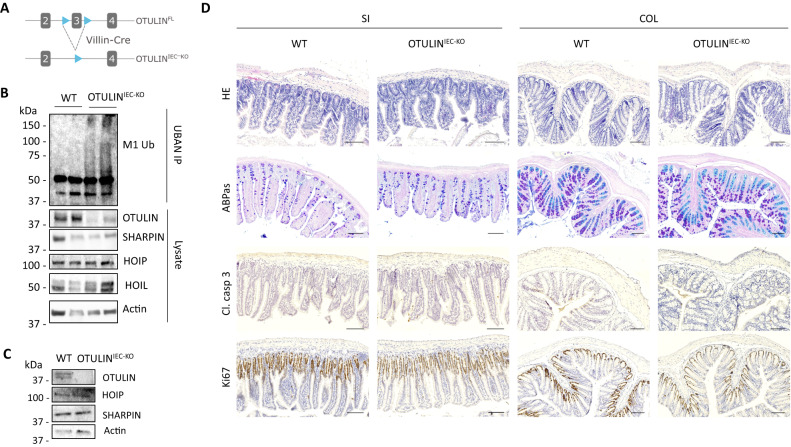
OTULIN^IEC-KO^ mice are healthy and do not develop intestinal pathology. **A** Targeting scheme showing the LoxP-flanked (floxed) and *Otulin* intestinal epithelial cell (IEC)-specific KO allele generated through Villin-Cre-mediated recombination. The boxes indicate exons, LoxP sites are indicated by arrowheads. **B** M1 chains were immunoprecipitated from IEC lysates of the small intestine of wild type (WT) and OTULIN^IEC-KO^ mice using GST-UBAN beads. Protein levels were determined by immunoblotting. Actin was used as a loading control. Data are representative of two independent experiments with two biological replicates. **C** Western blot analysis for the expression of OTULIN and LUBAC proteins in lysates of small intestinal organoids of wild type (WT) and OTULIN^IEC-KO^ mice. Actin was used as a loading control. Data are representative of two independent experiments. **D** Representative images of hematoxylin eosin (HE)-, Alcian blue/periodic acid–Schiff (ABPas)-, cleaved caspase-3- and Ki67-stained sections of small intestine (SI) and colon (COL) of 1-year-old WT (*n* = 6) and OTULIN^IEC-KO^ (*n* = 6) mice. Scalebar, 100 μm.

To investigate the role of OTULIN in the maintenance of the intestinal barrier, we generated mice that specifically lack OTULIN in the intestinal epithelium. To do this, mice with a floxed exon 3 of *Otulin* [14] were crossed with transgenic mice expressing *Cre* recombinase under control of the *Villin* promotor (Fig. 1A). The *Villin-Cre* transgenic line targets all epithelial cell lineages of the small intestine, cecum and colon, and expression mediates efficient Cre-mediated recombination in IECs starting before birth [29]. IEC-specific OTULIN knockout (*Otulin*^*FL/FL*^*Villin-Cre*, hereafter referred to as OTULIN^IEC-KO^) mice were born with normal Mendelian segregation (Suppl. Fig. 1A). OTULIN deletion was confirmed on protein level both in IEC lysates from the small intestine of OTULIN^IEC-KO^ mice as well as in small intestinal organoids derived from these mice (Fig. 1B, C). Western blot analysis also revealed that the expression levels of the LUBAC proteins HOIP, HOIL and SHARPIN are stable in IEC cells lacking OTULIN (Fig. 1B, C), which is similar to what was reported for bone marrow derived macrophages and THP-1 monocytes lacking OTULIN but different from what has been described for OTULIN-deficient B cells, T cells, fibroblasts, hepatocytes and keratinocytes [9, 10, 13–15]. Analysis of linear ubiquitination by specific UBAN pulldowns also confirmed the accumulation of linear polyubiquitin in IEC lysates of OTULIN-deficient mice (Fig. 1B), as previously shown in other OTULIN-deficient cell types [9, 11, 13–15, 30].

OTULIN^IEC-KO^ mice look healthy and have normal body weight (Suppl. Fig. 1B, C), and phenotypic analysis of OTULIN^IEC-KO^ mice up to the age of 12 months revealed no pathological signs in the intestine. Histologic analysis of tissue sections by hematoxylin-eosin staining showed normal morphology of both the small intestine and the colon of OTULIN^IEC-KO^ mice (Fig. 1D). Also, no difference in the number of goblet cells and Paneth cells could be observed between wild-type (WT) and OTULIN^IEC-KO^ mice, as revealed by Alcian-blue-Periodic acid Schiff (AB-PAS) staining (Fig. 1D). In agreement, expression analysis of Paneth cell-specific anti-microbial peptides (AMPs) *Cryptidin-1*, *Lysozyme-P, Reg3γ* and secreted *PLA2* and of goblet cell-specific *Muc2* by quantitative PCR on ileal epithelial cell lysates did not show any difference between WT and OTULIN^IEC-KO^ mice (Suppl. Fig. 2A). Next, since OTULIN was shown to prevent cell death [10, 11, 13–15, 30], we performed an immunostaining for cleaved caspase-3 to detect apoptotic cells. However, no differences could be observed in the number of apoptotic enterocytes between WT and OTULIN^IEC-KO^ mice, and only sporadically homeostatic apoptotic cells could be observed in the villus tip of the small intestines or at the top of the crypts in the colon of both lines (Fig. 1D). Also no differences were observed in expression of tight junction and desmosomal genes (*ZO-1, Claudin-4, Occludin, Desmoglein*) in intestinal lysates of WT and OTULIN^IEC-KO^ mice (Suppl. Fig. 2B). In addition, no differences in epithelial cell proliferation could be observed between WT and OTULIN^IEC-KO^ mice by Ki67 staining (Fig. 1D). Finally, we examined whether homeostatic NF-κB activation is affected by OTULIN deficiency in IECs by quantifying the expression of NF-κB-response genes (*I*κ*Bα, Tnfaip3, Muc2, Cryptidin-1*) in intestinal lysates of WT and OTULIN^IEC-KO^ mice. However, no significant differences in expression could be observed between both genotypes (Suppl. Fig. 2A, C). Together, these data demonstrate that intestinal epithelial OTULIN is dispensable for intestinal homeostasis in steady state conditions.

### OTULIN^IEC-KO^ mice are sensitized to experimental colitis

**Fig. 2:**
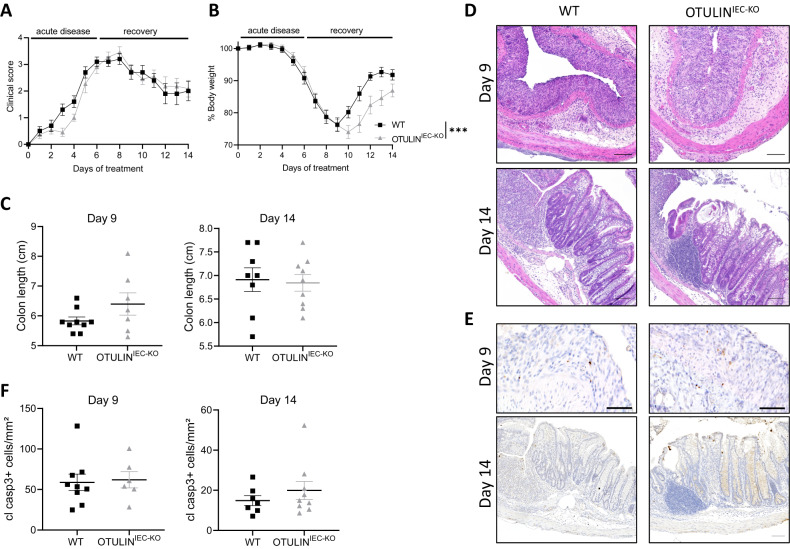
OTULIN^IEC-KO^ mice are sensitized to experimental colitis. **A**, **B** Clinical score (**A**) and body weight (**B**) of 9–15-week old female OTULIN^IEC-KO^ (*n* = 11) and control littermate (WT, *n* = 10) mice treated with 2% dextran sulfate sodium (DSS) for 6 days followed by normal drinking water. Data are represented as mean ± SEM, ***p < 0.001. Statistics represent differences in body weight between the genotypes over time. **C** Colon length of OTULIN^IEC-KO^ and control littermate mice, 9 (WT, *n* = 9; OTULIN^IEC-KO^, *n* = 7) and 14 days (WT, *n* = 8; OTULIN^IEC-KO^, *n* = 9) after the start of the experiment. Data are represented as mean ± SEM. **D** Representative images of hematoxylin eosin (HE) stained colon sections of OTULIN^IEC-KO^ and control (WT) littermate mice 9 and 14 days after the start of the experiment. Scalebar, 100 µm. **E** Representative images of cleaved caspase-3-stained colon sections of OTULIN^IEC-KO^ and control (WT) littermate mice 9 and 14 days after the start of the experiment. Scalebar, 100 μm. **F** Quantification of the number of cleaved caspase-3-positive cells on colon section 9 (WT, *n* = 9; OTULIN^IEC-KO^, *n* = 7) and 14 days (WT, *n* = 7; OTULIN^IEC-KO^, *n* = 9) after the start of the experiment. Data are represented as mean ± SEM.

Inflammatory bowel disease (IBD) is a chronic inflammatory disorder of the intestine of which the etiology is multifactorial and incompletely understood. One factor that can contribute to IBD development is an unstable mucosal barrier, allowing microbial infiltration and hyperactive immune responses towards the commensal microbiota [31]. To address whether OTULIN expression determines the susceptibility to IBD, OTULIN^IEC-KO^ mice and control WT littermates were evaluated in the established model of dextran sulfate sodium (DSS)-induced colitis. For this, mice were treated with 2% DSS in drinking water for 6 days followed by 8 days of regular drinking water, and were monitored daily for clinical pathology based on body weight loss, presence of fecal blood and stool consistency. Although OTULIN^IEC-KO^ mice and WT littermate control mice showed a similar disease progression with maximal clinical colitis on day 8–9 (Fig. 2A), OTULIN deficiency resulted in a slower recovery from colitis, as shown by the slower weight gain in the recovery phase of the experiment (Fig. 2B). However, no difference in colon length and colon histology could be detected (Fig. 2C, D), and no differences in the number of apoptotic cells could be observed by immunostaining for cleaved caspase-3 between WT and OTULIN^IEC-KO^ mice (Fig. 2E, F), at peak of disease (day 9) or at the end of the experiment (day 14). In conclusion, OTULIN^IEC-KO^ mice recover slower from DSS-induced colitis, suggesting that OTULIN is needed to reestablish the intestinal barrier stability upon acute inflammation.

### TNF induces IEC apoptosis and lethality in OTULIN^IEC-KO^ mice

**Fig. 3:**
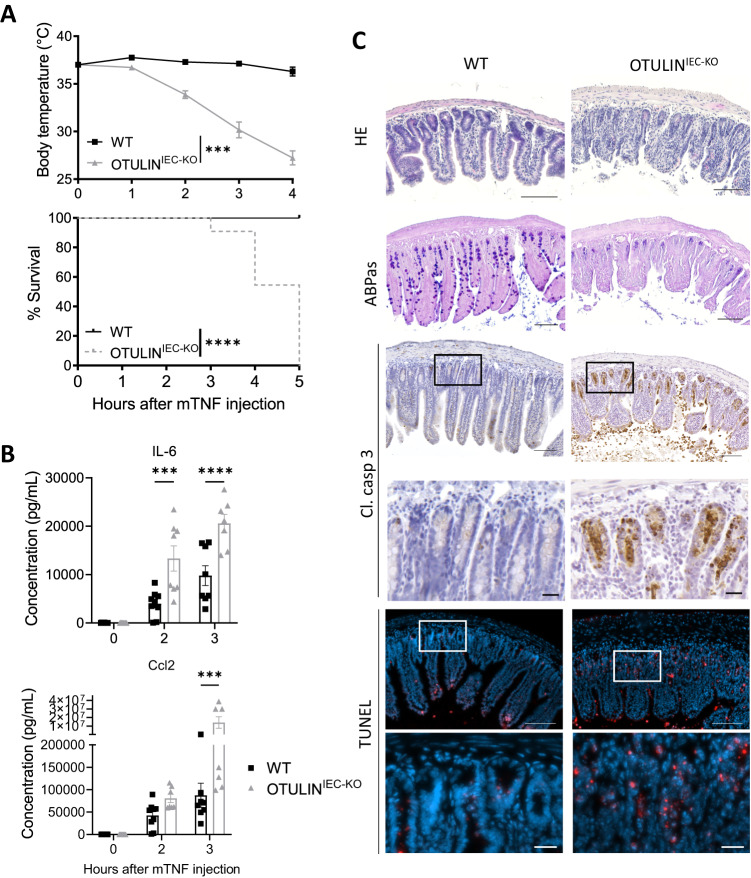
TNF induces IEC apoptosis and lethality in OTULIN^IEC-KO^ mice. **A**–**C** 8–15-week old OTULIN^IEC-KO^ and control (WT) littermate mice were injected intraperitoneally with 5 µg of recombinant mouse TNF per 20 g body weight. **A** Body temperature and survival of OTULIN^IEC-KO^ (*n* = 8) and control (WT, *n* = 11) mice. Data are represented as mean ± SEM, ***p < 0.001; ****p < 0.0001. Statistics represent differences in body weight or survival between the genotypes over time. **B** Serum IL-6 and Ccl2 levels in OTULIN^IEC-KO^ (t0, *n* = 10; t2, *n* = 8; t3, *n* = 7) and WT (t0 and t3, *n* = 7; t2, *n* = 9) mice. Data are represented as mean ± SEM, ***p < 0.001; ****p < 0.0001. **C** Representative images of hematoxylin eosin (HE)-, Alcian blue/periodic acid–Schiff (ABPas)-, cleaved caspase-3- and TUNEL-stained sections of the small intestine of 8–15-weeks old OTULIN^IEC-KO^ and control (WT) littermate mice 4 h after TNF injection. Scale bar overview, 100 μm; zoom, 20 μm.

TNF is a major proinflammatory cytokine that contributes to the pathogenesis of IBD in humans and experimental colitis in mice [26, 32]. Since OTULIN has been shown to regulate TNF signaling [33], we evaluated the impact of OTULIN deficiency in IECs on TNF-induced pathology. For this, we injected OTULIN^IEC-KO^ mice and control littermates with a normally sublethal dose of TNF. In contrast to control mice that only showed a modest drop in body temperature and all survived, OTULIN^IEC-KO^ mice were hypersensitive to TNF and showed a steep drop in body temperature already 2 h after TNF injection, and all died three to 5 h after TNF injection (Fig. 3A). This was accompanied by severe inflammation, as shown by high concentrations of the proinflammatory cytokines and chemokines, IL-6 and Ccl2, in serum of OTULIN^IEC-KO^ mice two and 3 h after TNF injection (Fig. 3B). Similarly, high expression of inflammatory mediators could be observed in IEC lysates of OTULIN^IEC-KO^ mice (Suppl. Fig. 3A). Immune profiling of myeloid cell populations using flow cytometry and immunostaining for F4/80 positive cells did not reveal major differences in infiltration of neutrophils, monocytes, macrophages, dendritic cells and eosinophils in the mucosa of the small intestine between both genotypes after TNF challenge (Suppl. Fig. 3B–D). However, histological analysis of the ileum showed extensive epithelial destruction, massive villus atrophy and reduced goblet cell numbers in the OTULIN^IEC-KO^ mice 4 h after TNF challenge, in contrast to control mice which maintain tissue integrity (Fig. 3C). We detected massive epithelial cell apoptosis both in the crypts and in the villi of OTULIN^IEC-KO^ mice by cleaved caspase-3 and TUNEL staining, while only limited cell death could be observed in the small intestine of WT mice (Fig. 3C). Increased apoptosis was also observed in the colon of OTULIN^IEC-KO^ mice after TNF injection, in contrast to the colon of WT mice where no apoptotic cells could be detected (Suppl. Fig. 4A, B). These observations suggest that TNF-driven breakdown of the intestinal barrier causes infiltration of commensal bacteria and systemic inflammation in OTULIN^IEC-KO^ mice.

**Fig. 4:**
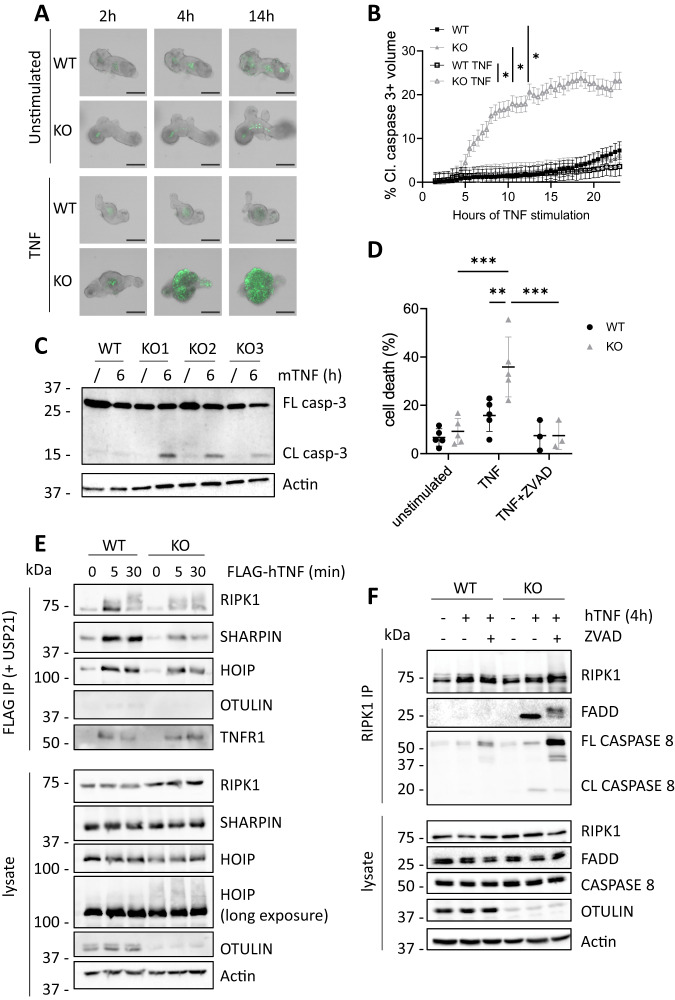
TNF induces IEC apoptosis in OTULIN deficient cells. **A** Representative images of organoids from the small intestine isolated from WT and OTULIN^IEC-KO^ mice which were left untreated or stimulated with 20 ng/mL mTNF for the indicated time points. Viability was assessed by caspase-3/7 green detection reagent. Scale bar, 100 μm. *n* = 3 biological replicates for each group and three technical replicates for each biological replicate. **B** Quantification of cell death as measured by caspase-3/7-positive signal in organoid cultures from WT and OTULIN^IEC-KO^ mice either or not stimulated with 20 ng/ml mTNF (*n* = 3 biological replicates for each group and three technical replicates for each biological replicate). Data are represented as mean ± SEM, *p < 0.05. Statistics represent differences in cell death between the genotypes over time. **C** Western blot analysis for full length (FL) and cleaved (CL) caspase-3 in lysates of small intestinal organoids isolated from OTULIN^IEC-KO^ (KO, *n* = 3 biological replicates and WT, *n* = 1) mice either stimulated or not stimulated with 20 ng/mL mTNF for 6 h. Actin was used as a loading control. **D** WT and OTULIN^KO^ HCT116 cells were treated with hTNF (20 ng/mL) for 24 h after which cell death was analysed by flow cytometry using propidium iodide and annexin V staining. Within the same condition, each dot is the result of a different experiment and is the mean of technical duplicates. **p < 0.01; ***p < 0.001. **E** WT and OTULIN^KO^ HCT116 cells were treated with 1.5 µg/ml FLAG-hTNF for the indicated timepoints. TNFR1 complex I was FLAG-immunoprecipitated and followed by USP21 treatment on the IPs. Protein levels were determined by immunoblotting. Actin was used as a loading control. Representative of two independent experiments. **F** WT and OTULIN^KO^ HCT116 cells were pretreated for 30 min with 50 µM ZVAD, as indicated, followed by 20 ng/mL hTNF for 4 h. RIPK1 was immunoprecipitated and protein levels were determined by immunoblotting. Actin was used as a loading control. Representative of two independent experiments.

In vitro studies confirmed the above findings in mice. Stimulation of intestinal organoids with TNF induced organoid disintegration and cell apoptosis only in the organoids derived from OTULIN^IEC-KO^ mice but not in organoids from WT mice (Fig. 4A–C). Similarly, OTULIN-deficient HCT116 cells were shown to be sensitized to TNF-induced apoptosis, which could be prevented in the presence of the pan-caspase inhibitor zVAD-fmk (Fig. 4D). When evaluating the effect of OTULIN deficiency on TNF receptor complex I formation following FLAG-TNF stimulation and FLAG immunoprecipitation, we could show an impaired recruitment of the LUBAC subunits HOIP and SHARPIN in OTULIN deficient HCT116 cells (Fig. 4E). OTULIN was not recruited to the TNFR1 signaling complex I following TNF stimulation (Fig. 4E), as previously shown [8]. These alterations coincide with enhanced formation of a cytosolic complex comprised of FADD and caspase 8, which is more abundant in OTULIN deficient HCT116 cells treated with TNF alone or with TNF and zVAD (Fig. 4F).

Finally, we assessed the impact of OTULIN deficiency on NF-κB and MAPK signaling. Although OTULIN deficiency enhances NF-κB and MAPK signaling downstream of nucleotide-oligomerization domain-containing protein 2 (NOD2), as previously shown [34], only minor differences could be observed in TNF-induced NF-κB and MAPK signaling between control and OTULIN deficient HCT116 cells (Suppl. Figure 5).

Together, these data demonstrate that OTULIN is critical to prevent IEC apoptosis in conditions of acute TNF exposure.

### OTULIN deficiency increases intestinal pathology in *TNF*^*emARE*^ mice

**Fig. 5:**
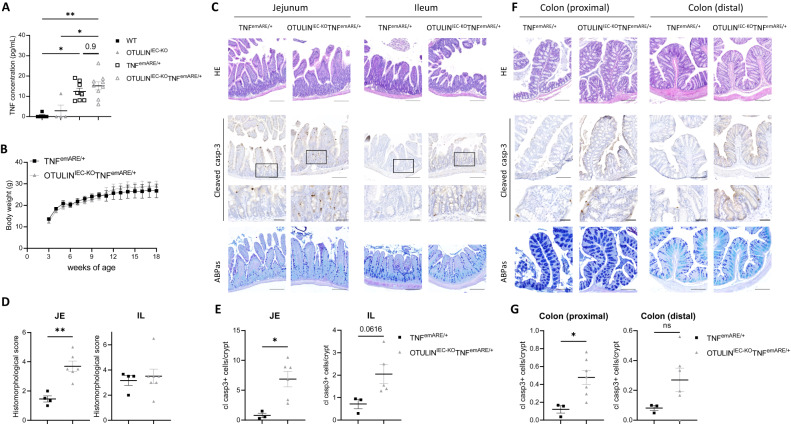
OTULIN deficiency increases intestinal pathology in TNF^emARE^ mice. **A** Serum TNF levels in 10-week old WT (*n* = 5), OTULIN^IEC-KO^ (*n* = 4), TNF^emARE/+^ (*n* = 8) and OTULIN^IEC-KO^ TNF^emARE/+^ (*n* = 9) mice. **B** Body weight of TNF^emARE/+^ (*n* = 3) and OTULIN^IEC-KO^ TNF^emARE/+^ (*n* = 9) mice. **C** Representative images of hematoxylin eosin (HE)-, cleaved caspase-3- and Alcian blue/periodic acid–Schiff (ABPas)-stained sections of the jejunum and ileum of 25-week old TNF^emARE/+^ and OTULIN^IEC-KO^ TNF^emARE/+^ mice. Scale bar, 200 μm; zoom, 50 μm. **D** Histomorphological pathology score of jejunum (JE) and ileum (IL) sections of 25-week old TNF^emARE/+^ (*n* = 4) and OTULIN^IEC-KO^ TNF^emARE/+^ (*n* = 7) mice. Data are represented as mean ± SEM, **p < 0.01. **E** Quantification of the number of cleaved caspase-3-positive cells in jejunum (JE) and ileum (IL) sections of TNF^emARE/+^ (*n* = 3) and OTULIN^IEC-KO^ TNF^emARE/+^ (*n* = 5-6) mice. Data are represented as mean ± SEM, *p < 0.05. **F** Representative images of hematoxylin eosin (HE)-, cleaved caspase-3- and Alcian blue/periodic acid–Schiff (ABPas)-stained sections of proximal and distal colon of 25-week old TNF^emARE/+^ and OTULIN^IEC-KO^ TNF^emARE/+^ mice. Scale bar, 200 μm; zoom, 50 μm. **G** Quantification of the number of cleaved caspase-3-positive cells in proximal and distal colon sections of TNF^emARE/+^ (*n* = 3) and OTULIN^IEC-KO^ TNF^emARE/+^ (*n* = 6) mice. Data are represented as mean ± SEM, *p < 0.05.

To investigate the importance of OTULIN in conditions of chronic TNF exposure, we crossed OTULIN^IEC-KO^ mice with TNF^emARE^ mice. TNF^emARE^ mice were generated by CRISPR/Cas9-mediated genome engineering, and lack the AU-rich elements (ARE) in the *Tnf* gene, leading to stabilization of *Tnf* mRNA and chronic elevated levels of TNF in the mice. Heterozygous TNF^emARE/+^ mice do not develop any spontaneous pathology, in contrast to homozygous TNF^emARE/ARE^ mice (unpublished data, Thiran et al., EMBO Mol. Med., in press), which phenocopy the previously published TNF^ΔARE^ mouse line [35] that spontaneously develop arthritis and ileitis. We confirmed the increased concentration of TNF in the serum of TNF^emARE/+^ mice compared to wild-type littermates, but TNF levels were not further increased upon OTULIN deletion (Fig. 5A). IEC-specific OTULIN deficiency did not affect body weight in TNF^emARE/+^ mice (Fig. 5B). However, histological analysis showed increased intestinal pathology in the jejunum of OTULIN^IEC-KO^ TNF^emARE/+^ mice compared to TNF^emARE/+^ mice (Fig. 5C, D). In contrast, no difference in disease development was observed in the ileum (Fig. 5C, D). Although cleaved caspase-3-positive apoptotic cells could be detected in the villi of TNF^emARE/+^ mice, significantly more cell death could be observed both in the crypts and the villi of the jejunum of OTULIN^IEC-KO^ TNF^emARE/+^ mice (Fig. 5C, E). A similar trend could be observed in the ileum of OTULIN^IEC-KO^ TNF^emARE/+^ mice compared to TNF^emARE/+^ mice, although not significant (p-value: 0.06) (Fig. 5C, E). Finally, we also addressed intestinal pathology in the colon, and observed increased numbers of cleaved caspase-3-positive apoptotic cells in OTULIN^IEC-KO^ TNF^emARE/+^ colon compared to the TNF^emARE/+^ condition (Fig. 5F, G).

Together, these data show that OTULIN also critically controls TNF-induced cell death in a transgenic model of chronic TNF-driven intestinal inflammation.

### OTULIN^IEC-KO^ mice are sensitized to *Salmonella*-induced colitis

**Fig. 6:**
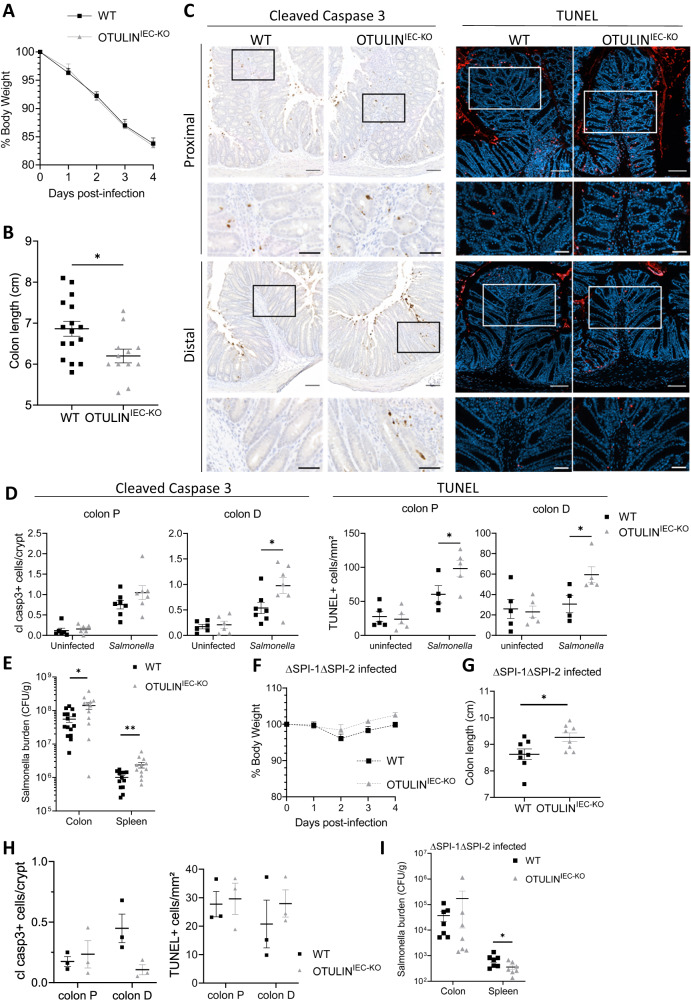
OTULIN^IEC-KO^ mice are sensitized to *Salmonella*-induced colitis. 8–11-week old control (WT, *n* = 18) and OTULIN^IEC-KO^ (*n* = 14) mice were infected with *Salmonella typhimurium* (A–E). **A** Percentage body weight of WT and OTULIN^IEC-KO^ mice after *Salmonella* infection. Data are represented as mean ± SEM. **B** Colon length of WT and OTULIN^IEC-KO^ mice 4 days post infection. Data are represented as mean ± SEM. *p < 0,05. **C** Representative images of cleaved caspase-3- and TUNEL-stained colon sections from WT and OTULIN^IEC-KO^ mice 4 days post infection. Scale bar, 100 μm; zoom 50 μm. **D** Quantification of the number of cleaved caspase-3- (WT and OTULIN^IEC-KO^ uninfected, *n* = 6; *Salmonella*, *n* = 7) and TUNEL- (WT and OTULIN^IEC-KO^ uninfected, *n* = 5; *Salmonella*, *n* = 4–5) positive cells in proximal (P) and distal (D) colon from WT and OTULIN^IEC-KO^ mice 4 days post infection. Data are represented as mean ± SEM. *p < 0.05. **E** *Salmonella* burden in the indicated tissue of WT and OTULIN^IEC-KO^ mice 4 days after infection. Data are represented as mean ± SEM. *p < 0,05; **p < 0,01. **F** Percentage body weight of WT (*n* = 10) and OTULIN^IEC-KO^ (*n* = 10) mice after ΔSPI-1ΔSPI-2 *Salmonella* infection. **G** Colon length of WT (*n* = 8) and OTULIN^IEC-KO^ (*n* = 8) mice 4 days post infection with ΔSPI-1ΔSPI-2 *Salmonella*. Data are represented as mean ± SEM. *p < 0.05. **H** Quantification of the number of cleaved caspase-3- and TUNEL-positive cells in proximal (P) and distal (D) colon from WT and OTULIN^IEC-KO^ mice (*n* = 3) 4 days post infection with ΔSPI-1ΔSPI-2 *Salmonella* . Data are represented as mean ± SEM. **I** *Salmonella* burden in the indicated tissue of WT (*n* = 7) and OTULIN^IEC-KO^ (*n* = 7) mice day 4 post infection with ΔSPI-1ΔSPI-2 *Salmonella*. Data are represented as mean ± SEM. *p < 0,05.

To complement our genetic and exogenous TNF supplementation models of OTULIN-dependent sensitivity, we evaluated colitis development in response to infection with the *Salmonella enterica* serovar Typhimurium (*Salmonella*) [36]. *Salmonella* infection induces TNF expression and hence provides an endogenous source of TNF within the intestinal tract [37]. Further, *Salmonella* infection leads to intestinal epithelial cell apoptosis, which acts as a nutrient source for bacteria [38]. OTULIN^IEC-KO^ and littermate control mice exhibited similar systemic morbidity during acute *Salmonella* infection as both genotypes lost body weight to a similar degree (Fig. 6A), and OTULIN^IEC-KO^ mice had similar cecal and spleen weights, and inflammatory gene expression in IECs as littermate controls (Suppl. Fig. 6A–C). However, OTULIN^IEC-KO^ mice showed a reduced colon length (Fig. 6B), suggesting that OTULIN deficiency might impact disease at the colonic tissue level. To assess if OTULIN-deficiency influenced cell death during *Salmonella* infection, we stained proximal and distal colon sections for cleaved caspase-3 or TUNEL. There was an increase in both apoptotic and general cell death, particularly in the distal colons of OTULIN^IEC-KO^ mice (Fig. 6C, D). Increased cell death correlated with exaggerated *Salmonella* burden in the colon and in bacterial dissemination to the spleen (Fig. 6E) but not in the small intestine or cecum (Suppl. Fig. 6D).

*Salmonella* uses two type-3 secretion systems encoded within the *Salmonella* pathogenicity island 1 (SPI-1) and SPI-2 that are largely responsible for inflammation and mammalian cell death in this model [38]. To test if OTULIN-dependent sensitivity requires these *Salmonella* virulence factors, we infected WT and OTULIN^IEC-KO^ mice with a mutant ΔSPI-1ΔSPI-2 strain. In contrast to fully virulent *Salmonella*, ΔSPI-1ΔSPI-2 *Salmonella* does not induce body weight loss (Fig. 6F). In the absence of SPI-1/SPI-2, OTULIN^IEC-KO^ mice had similar cecal and spleen weights (Suppl. Fig. 6E, F), did not show shortened colon length (Fig. 6G), nor increases in epithelial cell death (Fig. 6H, Suppl. Fig. 6G), or increased *Salmonella* burden compared to littermate controls (Fig. 6I, Suppl. Fig. 6H). Together, these findings demonstrate that OTULIN signaling within intestinal epithelial cells is critical for restricting cell death and pathogen burden in response to inflammation-inducing virulent *Salmonella* infection.

## Discussion

Lytic cell death is increasingly recognized as a major driver of inflammatory pathologies. Lytic forms of cell death, including necroptosis, pyroptosis, and secondary necrosis release DAMPs (danger-associated molecular patterns), that activate immune receptors and induce inflammatory responses. In contrast, apoptosis is commonly considered an anti-inflammatory cell death modality in most tissues, but excessive apoptosis in the intestinal epithelium can nonetheless promote inflammation due to loss of barrier integrity and the subsequent sensing of PAMPs (pathogen-associated molecular patterns) from commensal microbes that have breached the barrier [39]. Cells have developed multiple mechanisms to keep cell death in check to prevent the development of chronic inflammation, particularly at barrier sites. OTULIN has been identified as a critical regulator of cell death and inflammation in different organs such as liver and skin [11, 13–15, 30]. Humans with a homozygous loss-of-function mutation in *OTULIN* develop OTULIN-related autoinflammatory syndrome (ORAS), a potentially fatal, TNF-driven autoinflammatory disease characterized by severe systemic inflammation, recurrent fevers, panniculitis, and arthritis, but also with diarrhea [9, 10, 12], suggesting a role for OTULIN in intestinal homeostasis.

Although deletion of OTULIN in the intestinal epithelium of mice did not cause spontaneous intestinal pathology, we showed that these mice are hypersensitive to a TNF challenge, causing severe epithelial destruction and lethality. Indeed, OTULIN deficient IECs are shown to die from TNF-induced apoptosis. ORAS patients have high levels of circulating TNF with effects on multiple organs. To model the chronic systemic TNF condition in ORAS patients, we generated IEC-specific OTULIN deficient mice in the TNF^emARE/+^ background which spontaneously produce elevated levels of TNF. In response to chronic elevated TNF exposure, OTULIN^IEC-KO^ TNF^emARE/+^ mice develop elevated intestinal pathology and display enhanced epithelial cell death in the intestine. Analysis of intestinal biopsies of ORAS patients could reveal whether intestinal pathology in these patients is associated with increased IEC cell death.

The function of OTULIN is likely to prevent accumulation of M1-ubiquitin chains in the cytoplasm and prevent LUBAC autoubiquitination and degradation [8, 11]. Hence, OTULIN-deficient cells often show reduced expression of HOIP and SHARPIN, with enhanced sensitization to TNF-induced cell death [10, 11, 15, 30]. The intestinal epithelium of OTULIN^IEC-KO^ mice, however, shows normal expression levels of LUBAC proteins, questioning the mechanism of how OTULIN deficiency promotes epithelial cell death. Using HCT116 cells we demonstrated that, although LUBAC levels are unaffected in basal conditions, its recruitment to the TNF receptor signaling complex I in response to TNF stimulation is significantly impaired in OTULIN deficient conditions, promoting the formation of a cytosolic cell death-inducing signalling complex.

The importance of cell death for intestinal homeostasis and inflammatory bowel disease (IBD) has been extensively studied and demonstrated using gene-targeted knockout mice. Loss of proteins such as NEMO, RIPK1, caspase-8 and FADD in the intestinal epithelium causes spontaneous intestinal pathology due to aberrant activation of inflammatory cell death [16, 19–21]. OTULIN deletion in the IECs does not cause spontaneous pathology, however sensitizes mice to experimental colitis induced by exposure to DSS, and increases the susceptibility to *Salmonella* infection, confirming the importance of OTULIN for barrier integrity in conditions of inflammation and infection. Similar to the phenotype of OTULIN^IEC-KO^ mice, mice with intestinal epithelial deficiency in A20, a crucial ubiquitin-binding anti-inflammatory and cytoprotective protein that has been associated with IBD [40, 41], do not develop spontaneous intestinal pathology, but are also hypersensitive to TNF and DSS [18] and to infection with *Salmonella* [38]. In addition, a recent study showed that mice that lack the C-terminal catalytic center of HOIP, abolishing LUBACs E3 ligase activity, specifically in IECs are also normal but sensitized to TNF and DSS, indicating that linear ubiquitination in IECs protects against intestinal inflammation by suppressing TNF-induced IEC apoptosis under inflammatory conditions [42]. Together, our study identifies OTULIN as a major antiapoptotic protein in the intestinal epithelium that ensures maintenance of epithelial barrier integrity in inflammatory conditions, and suggest that OTULIN defects may increase susceptibility to IBD. However, so far, no genetic associations have been reported for OTULIN in IBD.

Finally, although OTULIN has been identified as a negative regulator of TNF-induced NF-κB activation [7, 9], especially in myeloid cell types, we did not observe a major inhibitory effect of OTULIN on homeostatic nor TNF-induced NF-κB signaling in IECs, in vivo nor in vitro, similar to earlier observations in fibroblasts, keratinocytes and hepatocytes [10, 11, 13–15, 30]. Based on these findings we can conclude that the intestinal phenotype of OTULIN^IEC-KO^ mice is caused by the hypersensitivity of the OTULIN-deficient epithelium to cell death affecting barrier integrity.

## Method details

### Mice

Mice with conditional loxP-flanked *Otulin* alleles [14] were crossed with *Villin*-Cre (B6.Cg-Tg(Vil1-cre)997Gum/J) mice [29]. TNF^emARE^ mice (B6J-Tnf^em1Irc^, unpublished) were generated by CRISPR-Cas9 gene targeting. For this, gRNA sequences 5′-GTGCAAATATAAATAGAGGG-3′ (gRNA1) and 5′-GGAAGGCCGGGGTGTCCTGG-3′ (gRNA2) were cloned in the BbsI site in the pX330 vector (addgene #42230). For sgRNA synthesis, the T7 promoter sequence was added to sgRNA forward primer and the IVT template was generated by PCR amplification using forward primers 5′-TTAATACGACTCACTATAGGTGCAAATATAAATAGAGGG-3′ and 5′- TTAATACGACTCACTATAGGGAAGGCCGGGGTGTCCTGG-3′ for gRNA1 and gRNA2, respectively, and reverse primer 5′-AAAAGCACCGACTCGGTGCC-3′. The T7-sgRNA PCR product was purified and used as the template for IVT using MEGAshortscript T7 kit (Thermofisher). Both sgRNAs were purified using the MEGAclear kit (Thermofisher). B6J-Tnf^em1Irc^ mice were generated by injecting a mix of gRNA1 (10 ng/µl), gRNA2 (10 ng/µl), Cas9 protein (40 ng/µl; VIB Protein Service Facility) and Cas9 mRNA (20 ng/µl, Thermofisher) in C57BL/6J zygotes. Injected zygotes were incubated overnight in Embryomax KSOM medium (Merck, Millipore) in a CO2 incubator. The following day, 2-cell embryos were transferred to pseudopregnant B6CBAF1 foster mothers. The resulting pups were screened by PCR over the target region using primers 5′-TCTCATGCACCACCATCAA-3′ and 5′-GCAGAGGTTCAGTGATGTAG-3′. PCR bands were Sanger sequenced to identify the exact nature of the deletion. B6J-Tnf^em1Irc^ contains an allele with a deletion of 107 bp in the 3′ UTR of the *Tnf* gene at Chromosome 17:35418603-35418709 (GRCm39). This mutation is predicted to cause stabilization of the *Tnf* mRNA.

All alleles were maintained on a C57BL/6 J genetic background. Mice were housed in individually-ventilated cages at the VIB Center for Inflammation Research, in a specific pathogen-free animal facility. All experiments on mice were conducted according to institutional, national and European animal regulations. Animal protocols were approved by the ethics committee of Ghent University (permit number LA1400091).

### Isolation of intestinal crypts and three-dimensional organoid culture

Intestinal organoids were derived from the small intestine, as previously described [43]. Briefly, a 10 cm piece of duodenum/jejunum was dissected and washed in phosphate-buffered saline (PBS). The intestine was opened longitudinally, villi were scraped away and the tissue was chopped in 2–3 mm pieces. After thorough washing in PBS, pieces were incubated in 2 mM EDTA/PBS for 30 min at 4 °C on a rocking platform. The pieces were shaken vigorously in 2% fetal calf serum and after passage through a 70-μm cell strainer, crypt fractions were isolated and purified by successive centrifugation steps. One ml of matrigel (BD Biosciences) was added to a pellet of 100–1000 crypts, and drops of 50 μl crypt-containing matrigel were added to pre-warmed wells in a 24-well plate. After polymerization, 500 μl complete growth medium (advanced DMEM/F12, Gibco) containing EGF (50 ng/mL, Invitrogen), R-Spondin1 (from conditioned medium from 293T-HA-RspoI-Fc cells), Noggin (from conditioned medium), N2 (Gibco), B2 (Gibco), Hepes (10 mM, Invitrogen), Glutamax (Invitrogen), N-Acetylcycsteine (1 mM, Sigma) and 1% penicillin/streptomycin (Gibco) [44] was added to each well every 2 days. For TNF stimulation (*Escherichia coli*-derived recombinant protein produced by VIB-Protein service facility), 20 ng/mL mTNF was diluted in organoid growth medium.

### OTULIN deficient HCT116 cells

OTULIN-deficient HCT116 cells were generated via CRISPR–Cas9 gene targeting. In short, a sgRNA was designed using the CRISPRscan tool (OTULIN: 5′ CACCGGTGCGCCGAGACGCCGGCG 3′) and cloned into the px458 vector (Addgene plasmid, 48138). 10^6^ cells were electroporated with 10 μg DNA using the NEPA21 cuvette electroporator. Two days after electroporation, GFP-positive single cells were sorted in a 96-well plate. Finally, clones were expanded and screened using Western blot analysis, to select the desired OTULIN-knockout clones. The cells tested negative for mycoplasma. Cells were cultured in complete DMEM (Gibco) supplemented with 10% fetal calf serum (Bodinco), 1% penicillin/streptomycin (Gibco), 1% L-Glutamine (Lonza), 0,4% sodium-pyruvate (Sigma) and 1% non-essential amino acids (Lonza). Cells were left untreated or stimulated with hTNF (20 ng/mL, VIB Protein Service Facility), ZVAD (50 µM, Enzo Life Sciences) or L18-MDP (1 µg/ml, Invivogen, tlrl-lmdp) for the indicated time points.

### PI-Annexin cell death analysis

After induction of cell death, cells were stained with annexin V-APC (BioLegend, Cat# 640941, 1:50) and propidium iodide (Sigma, Cat# 4170,2 µg/mL) for 15 min at room temperature in annexin V binding buffer (BD). Flow cytometry analysis was performed using the FACS Calibur (BD) and LSR II HTS (BD). Data were analysed with FlowJo v.10 software.

### Live cell imaging

Small intestinal organoids were isolated as described above and seeded in μ-slide 8 well (iBidi). CellEvent™ Caspase-3/7 Green Detection Reagent (Invitrogen) was added to the medium according to the manufacturer’s instructions. Three organoids per condition (technical triplicates) were followed over time at time intervals of 30 min and z-stack images were made using the Zeiss spinning disk. Images were processed using ImageJ. For each image, the total volume of green signal was determined and normalized to the total volume of the organoid.

### IEC isolation

Small intestines or colons were dissected and flushed with cold PBS to remove fecal contents. The intestine was turned inside out, washed in cold PBS and incubated in 2 mM EDTA/HBSS at 37 °C for 20 min. Next, the tubes were shaken vigorously and the supernatant containing the IECs was further processed for Western blot or qPCR.

### Western blot analysis

Organoids and IECs were homogenized using E1A lysis buffer (50 mM HEPES pH7.6; 250 mM NaCl; 5 mM EDTA; 0.5% NP40) and RIPA (50 mM Tris-HCl, pH 7.6; 1 mM EDTA; 150 mM NaCl; 1% NP-40; 0.5% sodiumdeoxycholate; 0.1% SDS) buffer, respectively containing protease inhibitors (Roche) and phosphatase inhibitors (Sigma), denaturated in 1 × Laemmli buffer (50 mM Tris-HCl pH8.2; 2% SDS; 10% glycerol; 0.005% BFB; 5% β-mercapto-ethanol) and boiled for 10 min at 95 °C. 50 µg of IEC lysates and 20 µg of organoid lysates were separated by SDS-polyacrylamide gel electrophoresis (PAGE), transferred to nitrocellulose and analysed by immunoblotting. Membranes were probed with antibodies against OTULIN (1:1000, Cell Signaling Technology, Cat# 14127), JNK (1:1000, BD Bioscience, Cat# 554285), phospho-JNK (1:1000, Millipore, Cat# PS1019), IκBα (1:1000, Cell Signaling, Cat# 9242), phospho-IκBα (1:1000, Cell Signaling Technology, Cat# 9246), p38 MAPK (1:1000, Cell Signaling Technology, Cat# 9212), phospho-p38 MAPK (1:1000, Cell Signaling Technology, Cat# 9215), caspase-3 (full length and cleaved) (1:1000, Cell Signaling Technology, Cat# 9662), HOIL-1 (1:2000, kind gift of Dr. Henning Walczak, UCL London), HOIP (1:1000, kind gift of Dr. Rune Damgaard, MRC Cambridge), HOIP (1:1000, Abcam, Cat# ab46322), Sharpin (1:1000, Proteintech, Cat# 14626-1-AP), linear ubiquitin (1:2500, LifeSensor, Cat# AB130), K63 ubiquitin (1:1000, Merck, Cat# 05-1308), RIPK1 (1:1000, Cell signaling, Cat# 3493), TNFR1 (1:1000, Santa Cruz, Cat# sc-8436), FADD (1:500, Enzo Life Sciences, Cat# ADI-AAM-212-E), Caspase 8 (1:1000, Prof. P. Krammer), and actin-HRP (1:10000, MP Biomedicals, Cat# sc-47778 HRP). As secondary antibodies, HRP-coupled anti-rabbit-HRP (1:2500, GE Healthcare, Cat# GENA934) and anti-mouse-HRP (1:2500, GE Healthcare, Cat# NA931) were used and detection was done by chemiluminescence (Western Lightning Plus ECL, Perkin Elmer) using the Amersham Imager 600 (GE Healthcare).

### Immunoprecipitation

Recombinant GST-UBAN was produced in BL21(DE3) cells. In brief, BL21(DE3) cells were transformed with the plasmid encoding GST-UBAN and protein expression was induced with 0.5 M IPTG. After 4 h, cells were collected and lysed in lysis buffer (20 mM Tris-HCl pH 7.5, 10 mM EDTA, 5 mM EGTA, 150 mM NaCl, 1 mM DTT supplemented with phosphatase and protease inhibitor cocktail tablets (Roche Diagnostics)), sonicated and cleared by centrifugation. After centrifugation, Triton-X100 (0.5% final concentration) was added to the supernatant, which was then transferred onto prewashed glutathione beads and left rotating for 2 h at 4 °C. After incubation, the beads were centrifuged, washed twice with washing buffer (20 mM Tris-HCl pH 7.5, 10 mM EDTA, 150 mM NaCl, 0.5% Triton-X100) and resuspended in resuspension buffer (20 mM Tris-HCl pH 7.5, 0.1% β-mercaptoethanol, 0.05% sodiumazide), ready to be used. Cell lysates from IECs were prepared as described before, protein concentration was determined and 2 mg protein lysate was incubated overnight with GST-UBAN-containing glutathione beads. The beads were washed three times in RIPA lysis buffer (150 mM NaCl, 1% NP-40, 0.5% Sodium Deoxycholate, 0.1% SDS, 10 mM Tris-HCl pH 8 supplemented with phosphatase (Sigma) and protease (Roche Diagnostics) inhibitor cocktail tablets), and resuspended in 60 μL 1x laemmli buffer for direct analysis. For immunoprecipitation of complex I, HCT116 cells were stimulated with 1.5 μg/ml Flag-hTNF (VIB Protein Service Facility). Cells were then washed 2 times in ice-cold PBS and lysed in 1 ml NP-40 lysis buffer (10% glycerol, 1% NP-40, 150 mM NaCl and 10 mM Tris-HCl pH 8, supplemented with phosphatase and protease inhibitor cocktail tablets (Roche Diagnostics)). The cell lysates were cleared by centrifugation for 15 min at 4 °C and the supernatants were then incubated overnight with FLAG M2 affinity gel (Sigma-Aldrich). Receptor complex proteins were eluted from the beads using 200 ng/mL 3× FLAG peptide (Sigma) as described in the manufacturer’s instructions. Protein complexes were additionally deubiquitylated by incubation of beads with 1.2 μg USP21 (VIB Protein Service Facility) in DUB buffer (50 mM Tris-HCl pH8, 50 mM NaCl, 5 mM DTT and 1 mM MnCl2) for 30 min at 37 °C before FLAG peptide elution. For immunoprecipitation of complex II, HCT116 cells were pretreated with ZVAD (50 µM, Enzo Life Sciences) for 30 min, followed by hTNF (20 ng/mL) stimulation for the indicated timepoints. The cells were lysed in NP40 lysis buffer and lysates were cleared by centrifugation and the supernatants were incubated with RIPK1 antibody (Cell signaling, Cat# 3493) and Protein A Sepharose beads (Cytiva, GE17-5280-01).

### In vivo TNF toxicity

Mice were injected intraperitoneally with a sublethal dose of mouse TNF (5 μg mouse TNF/20 g body weight). *E. coli*-derived recombinant mTNF had a specific activity of 9.46 ×10^7^ IU/mg, and was produced and purified to homogeneity in our laboratory with endotoxin levels not exceeding 1 ng/mg protein. Body temperature and survival were monitored every hour. Mice were euthanized on the indicated timepoints.

### DSS-induced colitis

9–15-week old OTULIN^IEC-KO^ and littermate WT controls were used in DSS-induced colitis experiments. Acute colitis was induced by addition of 2 % DSS (36–50 kD; MP Biomedicals) to the drinking water for 6 days followed by regular distilled water. Body weight, occult or gross blood loss per rectum, and stool consistency were determined daily during the colitis experiment. Fecal blood was determined using Hemoccult SENSA (Beckman Coulter) analysis. The baseline clinical score was determined on day 0. In brief, no weight loss was scored as 0, weight loss of 1–5% from baseline as 1; 5–10% as 2; 10–20% as 3; and >20% as 4. For bleeding, a score of 0 was assigned for no blood, 2 for positive hemoccult, and 4 for gross bleeding. For stool consistency, a score of 0 was assigned for well-formed pellets, pasty and semiformed stools were scored as 2, and liquid stools as 4. The average of these scores resulted in a total clinical score ranging from 0 (healthy) to 4 (maximal colitis). To eliminate any diagnostic bias, mice were scored blindly.

### Salmonella-induced colitis

Littermate-, sex- and age-matched mice were infected using the *Salmonella enterica* serovar Typhimurium (strain SL1344 or ΔSPI-1ΔSPI-2) model of colitis [36]. SPF mice were given a single dose of 20 mg streptomycin via oral gavage (100 μl of 200 mg ml^−1^ streptomycin dissolved in water) one day before infection. Mice were infected with 1 × 10^7^ CFU per mouse resuspended in 1× PBS via oral gavage. Mouse body weight was measured daily and the percentage body weight was calculated as (daily weight)/(day 0 starting weight). At the indicated day after infection, intestinal tissue (ileum, cecum and colon) and the spleen were collected. For bacterial burden measurements and histology, luminal contents of the ileum (the last 5–6 cm of the distal end of the small intestine) and the colon were removed. Luminal content was retained in all cecal samples. Any attaching lymphatic tissue was removed from intestinal tissue before homogenization in 1 ml of 1× PBS. CFU per gram of tissue were calculated by plating serial dilutions of tissue homogenates on MacConkey agar containing streptomycin and MacConkey agar containing streptomycin and kanamycin. Tissue samples were excluded if the total burden fell below the limit of detection. Single strain infection tissue homogenates were plated solely on MacConkey agar containing streptomycin.

### Histology

Small intestine and colon were dissected, fixed in 10 % formalin and embedded in paraffin. Sections were stained with Hematoxylin/Eosin (HE) or with specific stains or antibodies. For immunohistochemistry, following dewaxing, slides were incubated in antigen retrieval solution (Dako), boiled for 20 min in a PickCell cooking unit and cooled down for 3 h. Endogenous peroxidase activity was blocked by incubating the slides in 3% wv H_2_O_2_ (Sigma). The blocking buffer contains 0.2% goat serum, 0.5% Fish skin gelatin and 2 % BSA in PBS. Subsequently, slides were incubated with anti-cleaved caspase 3 (1:300, Cell Signaling, Cat# 9661), anti-Ki67 (1:1000, Cell Signaling, Cat# 12202), or F4/80 (1:50, BioRad, Cat# MCA497G) primary antibodies overnight at 4 °C. Next, the slides were incubated with biotin coupled goat anti-rabbit (1:500, Dako, Cat# E0432), and incubated in ABC solution (Vectastain ELITE ABC Kit Standard, Vector Laboratories). Peroxidase activity was detected by adding diaminobutyric acid (DAB) substrate (ImmPACT DAB Peroxidase (HRP) Substrate, Vector Laboratories), and slides were counterstained with hematoxylin (Sigma), dehydrated and mounted with Entellan (Merck). For the F4/80 staining, a similar protocol was followed, without antigen retrieval and using 5% goat serum in 1% BSA as blocking buffer, and biotin coupled goat anti-rat (1:500, Vector Laboratories, Cat# BA-9401) as secondary antibody. TUNEL kit (Millipore) was used to visualize death cells, according to manufacturer’s instructions. For combined Alcian Blue and Periodic Acid-Schiff stainings (AB/Pas), dewaxed sections were incubated in AB for 20 min. Sections were subsequently washed with water before incubation in 1% periodic acid for 10 min followed by incubation in Schiff’s reagent for 10 min. Sections were counterstained with Mayer’s haematoxylin for 30 s, washed and dehydrated before mounting with Entellan (Merck). The pictures were taken with the Zeiss slide scanner. To eliminate any diagnostic bias, quantification of cleaved caspase 3 and TUNEL stainings was done blindly.

### Histomorphologyical score of TNF^emARE^ sections

HE sections of TNF^emARE^ mice were scored blindly according to following scoring scheme: for goblet cells a score of 0 was given if the amount of goblet cells is normal, 1 for minimal goblet cell loss (1–30 %), 2 for obvious goblet cell loss (30–60 %), 3 for severe goblet cell loss (60–90 %) and 4 for maximal goblet cell los (>90%). For immune cell infiltration a score of 0 represents no immune cell infiltration, 1 immune cell infiltration in the lamina propria, 2 infiltration in the mucosa, 3 infiltration in the submucosa and 4 transmural infiltration. Finally, villus architecture was scored as 0 for normal structures, 1 for minimal villus blunting, 2 for blunted villi with only 50% of crypt remaining, 3 for blunted villi with only 25% of crypt remaining and 4 for maximal blunting and loss of callus-crypt architecture. The three scores were summed resulting a score ranging from 0 (normal) to 12 (maximal pathology).

### Quantification of cytokines

Serum was isolated from adult mice and IL-6 and Ccl2 was measured by Bio-Plex (Biorad), according to manufacturer’s instructions. TNF levels were determined by ELISA (Mouse Uncoated ELISA Kit with Plates, Invitrogen) according to manufacturers’ instructions.

### Quantitative real-time PCR

Total RNA was isolated using TRIzol reagent (Invitrogen) and Aurum Total RNA Isolation Mini Kit (Biorad), according to manufacturer’s instructions. Synthesis of cDNA was performed using Sensifast cDNA Synthesis Kit (Bioline) according to the manufacturer’s instructions. cDNA was amplified on quantitative PCR in a total volume of 5 µl with SensiFAST SYBR® No-ROX Kit (Bioline) and specific primers on a LightCycler 480 (Roche). The reactions were performed in triplicates. Mouse-specific primers that were used for this study: *Il6* forward 5′ GAGGATACCACTCCCAACAGACC 3′, reverse 5′ AAGTGCATCATCGTTGTTCATACA 3′; *Tnf* forward 5′ ACCCTGGTATGAGCCCATATAC 3′, reverse 5′ ACACCCATTCCCTTCACAGAG 3′; *Ccl2* forward 5′ TTAAAAACCTGGATCGGAACCAA 3′, reverse 5′ GCATTAGCTTCAGATTTACGGGT 3′; *Ccl5* forward 5′ CGTCAAGGAGTATTTCTACAC 3′, reverse 5′ GGTCAGAATCAAGAAACCCT 3′; *Il1b* forward 5′ CAACCAACAAGTGATATTCTCCATG 3′, reverse 5′ GATCCACACTCTCCAGCTGCA 3′; *Tnfaip3* forward 5′ AAACCAATGGTGATGGAAACTG 3′, reverse 5′ GTTGTCCCATTCGTCATTCC 3′; *Ikba* forward 5′ GTAACCTACCAAGGCTACTC 3′, reverse 5′ GCCACTTTCCACTTATAATGTC 3′; *Muc2* forward 5′ ATGACACCATCTACCTCACC 3′, reverse 5′ ACTGAACTGTATGCCTTCCTC 3′; *ZO-1* forward 5′ TGGAATTGCAATCTCTGGTG 3′, reverse 5′ TTTTCCTGTAGCTGTCCTTCA 3′; *Claudin-4* forward 5′ ACCCACCCACCTACCCTACTA 3′, reverse 5′ TCCCCAGCCCTCCCCAAACCA 3′; *Dsg2* forward 5′ CCTTTCGGCATATTCGTCTTT 3′, reverse 5′ AGTCCAATGCATAGCCTGTCA 3′; *Occludin* forward 5′ CTGTGAAAACCCGAAGAAAGA 3′, reverse 5′ ATGTCCAGGCTCCCAAGATAA 3′; *LysP* forward 5′ GCCAAGGTCTAACAATCGTTGTGAGTTG 3′, reverse 5′ CAGTCAGCCAGCTTGACACCACG 3′; *Crypt-1* forward 5′ TCAAGAGGCTGCAAAGGAAGAGAAC 3′, reverse 5′ TGGTCTCCATGTTCAGCGACAGC 3′; *sPLA2* forward 5′ AGGATTCCCCCAAGATGCCAC 3′, reverse 5′ CAGCCGTTTCTGACAGGAGTTCTGG 3′; *Reg3g* forward 5′ CCTCAGGACATCTTGTGTCTGTGCTC 3′, reverse 5′ TCCACCTCTGTTGGGTTCATAGCC 3′; *Gapdh* forward 5′ TGAAGCAGGCATCTGAGGG 3′, reverse 5′ CGAAGGTGGAAGAGTGGGAG 3′; *Oaz1* forward 5′ AGAGACGCAGCGGAGGTTTT 3′, reverse 5′ TCTGGCGAAGCAGTGGCTAT 3′.

### Flow cytometry analysis

Lamina propria isolation from the small intestine of mice was performed by two treatments with HBSS + 2 mM EDTA, followed by treatment with collagenase VIII (0,6 mg/ml). The isolated cells (1 × 10^6^ cells per sample) were analyzed by flow cytometry for myeloid markers. The separation of alive/dead cells was possible by staining with the Fixable Viability Dye eFluor506 (eBioscience, Cat# 65-0866-14). The samples were further stained with the following antibodies: anti-CD45 AlexaFluor 700 (eBioscience, Cat# 56-0451-82), anti-SiglecF BUV395 (BD, Cat# 740280), anti-Ly6G PercpCy5.5 (BD, Cat# 560602), anti-Ly6C APC (eBioscience, Cat# 17-5932-80), anti-CD11b BV605 (BD, Cat# 563015), anti-CD64 BV711 (BioLegend, Cat# 139311), anti-F4/80 Biotin (eBioscience, Cat# 13-4801-82), Streptavidin BV421 (BioLegend, Cat# 405226), anti-CD11c PE-eFluor 610 (eBioscience, Cat# 61-0114-82) and anti-MHC ClassII APC-eFluor780 (eBioscience, Cat# 47-5321-80), lineage [CD19 (eBioscience, Cat# 15-0193-82), CD3 (eBioscience, Cat# 15-0031-82) and NK1.1 (BioLegend, Cat# 108716) PE-Cy5]. The cells were later fixed and processed for analysis using a five-laser BD LSRFortessa. The flow cytometry data were analyzed using FlowJo software 10.8.1 and presented as percentage to CD45+ population.

### Quantification and statistical analysis

Results are expressed as the mean ± SEM (unless stated otherwise), with n the number of mice. Outliers were identified using graphpad (ROUT, Q = 1%) and excluded from analysis. Normality tests (Kolmogorov-Smirnov) were performed to check for normal distribution of the data. Parametric tests were performed on normally distributed data, and non-parametric tests on data that were not normally distributed. Statistical significance between WT and OTULIN^IEC-KO^ was assessed using an unpaired two-sided Student t-Test, or non-parametric Mann-Whitney test. Statistical significance between WT and OTULIN^IEC-KO^ at different time points or between untreated and *Salmonella* infected was assessed using a two-way ANOVA test, followed by Sidák’s multiple comparison test. A Kruskal-Wallis test was performed for the analysis between WT, OTULIN^IEC-KO^, TNF^emARE^ and OTULIN^IEC-KO^TNF^emARE^ mice followed by a Dunn’s multiple comparison test. Survival of mice after injection with TNF was analysed using a Mantel-Cox test. All above analysis were performed in the Prism software (V9). Statistical details can be found in the figure legends. Body temperature, body weight, clinical disease score and organoid live cell imaging data followed over time were analysed as repeated measurements using method of residual maximum likelihood (REML), as implemented in Genstat version 20. Briefly, a linear mixed model (random terms underlined) of the form y = genotype + time + genotype × time + mouse × time was fitted to the longitudinal data. The term mouse × time represents the residual error term with dependent errors because the repeated measurements are taken in the same individual, causing correlations among observations. The autoregressive correlation model (AR) was finally selected as best fitted model based on the Akaike’s information criterion coefficient. The AR covariance model assumes that correlation between observations decays as the measurements are collected further apart in time. Additional options selected to get a best fitting model included 1) times of measurement were set as equally spaced, and 2) allowance of unequal variances across time. The significance of the fixed main and interaction terms in the model were assessed using an approximate F-test as implemented in Genstat version 20 [45].

## Supplementary information

Supplementary figures and figure legends

uncropped western blot file

## Data Availability

All datasets generated and analysed during this study are included in this published article and its Supplementary Information files. Additional data are available from the corresponding author on reasonable request.
