# Supplementary material for: OTULIN protects the intestinal epithelium from apoptosis during inflammation and infection
Source: Cell Death Dis. 2023 Aug 19;14(8):534. doi: 10.1038/s41419-023-06058-7 (PMC10439912; doi:10.1038/s41419-023-06058-7)
Supplement: Supplementary file 1 — Supplementary figures and figure legends [file 41419_2023_6058_MOESM1_ESM.pptx]

## Slide 1
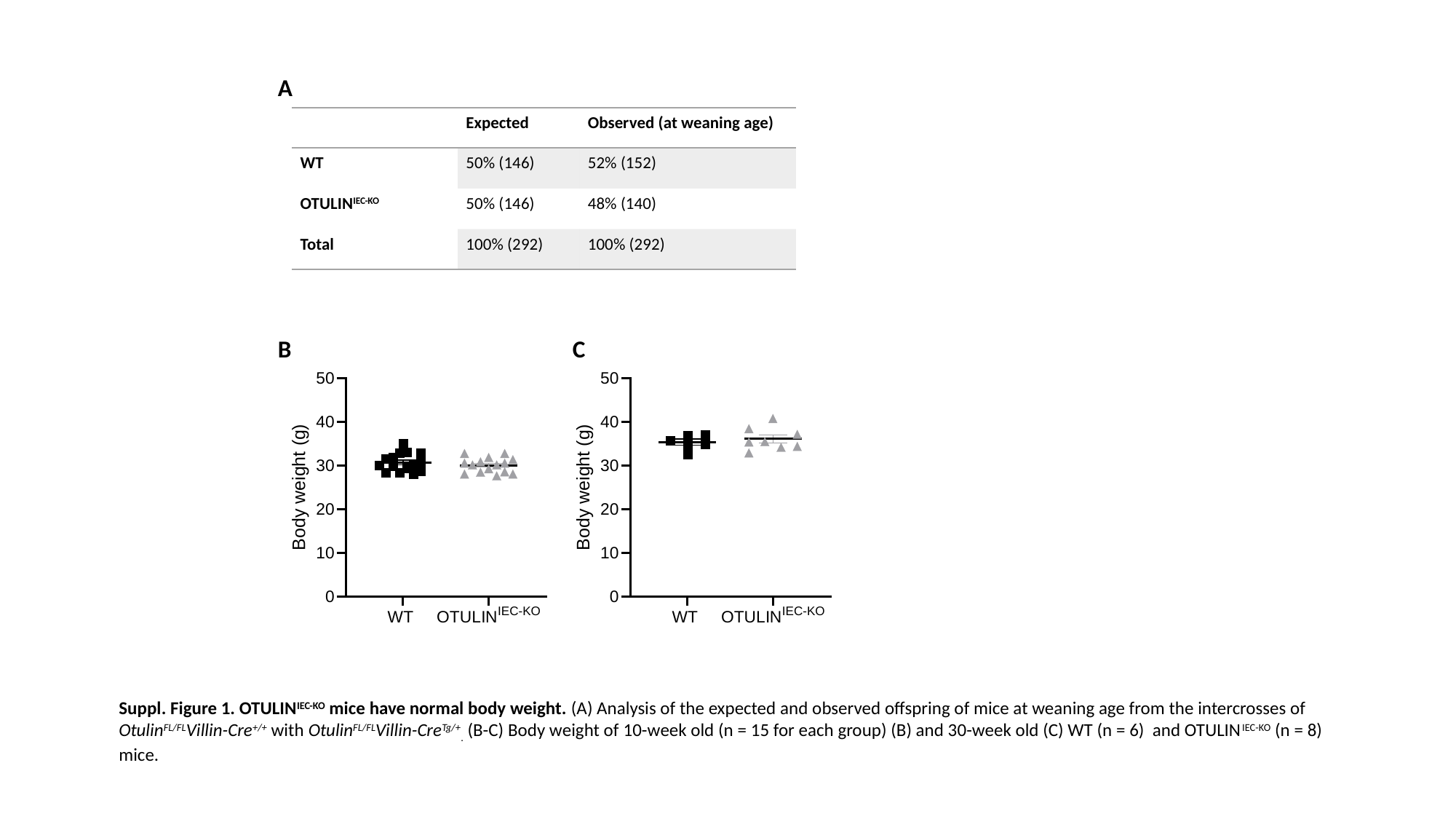

A
| | Expected | Observed (at weaning age) |
| --- | --- | --- |
| WT | 50% (146) | 52% (152) |
| OTULINIEC-KO | 50% (146) | 48% (140) |
| Total | 100% (292) | 100% (292) |
B
C
Suppl. Figure 1. OTULINIEC-KO mice have normal body weight. (A) Analysis of the expected and observed offspring of mice at weaning age from the intercrosses of OtulinFL/FLVillin-Cre+/+ with OtulinFL/FLVillin-CreTg/+. (B-C) Body weight of 10-week old (n = 15 for each group) (B) and 30-week old (C) WT (n = 6) and OTULINIEC-KO (n = 8) mice.

## Slide 2
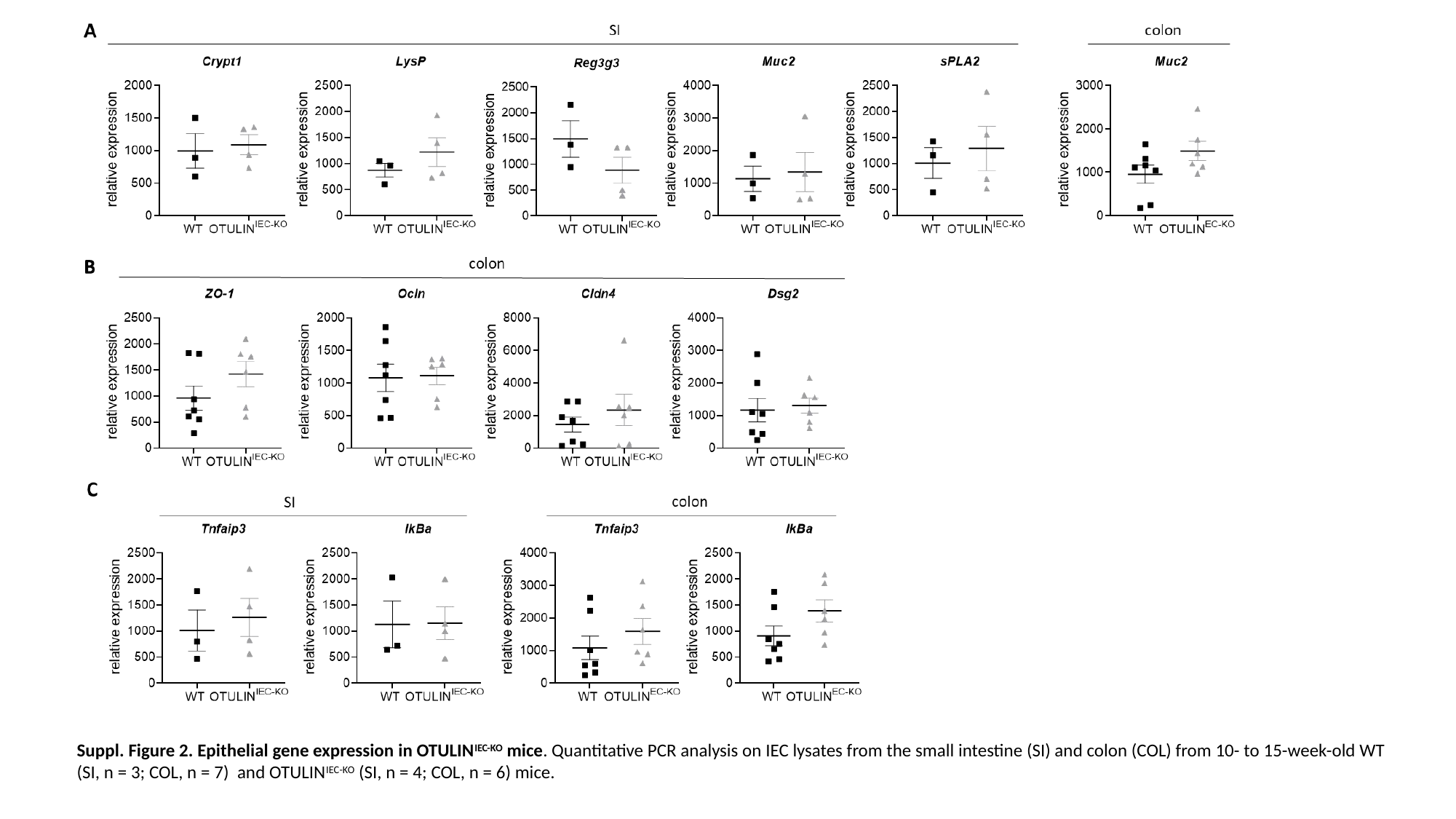

Suppl. Figure 2. Epithelial gene expression in OTULINIEC-KO mice. Quantitative PCR analysis on IEC lysates from the small intestine (SI) and colon (COL) from 10- to 15-week-old WT (SI, n = 3; COL, n = 7) and OTULINIEC-KO (SI, n = 4; COL, n = 6) mice.

## Slide 3
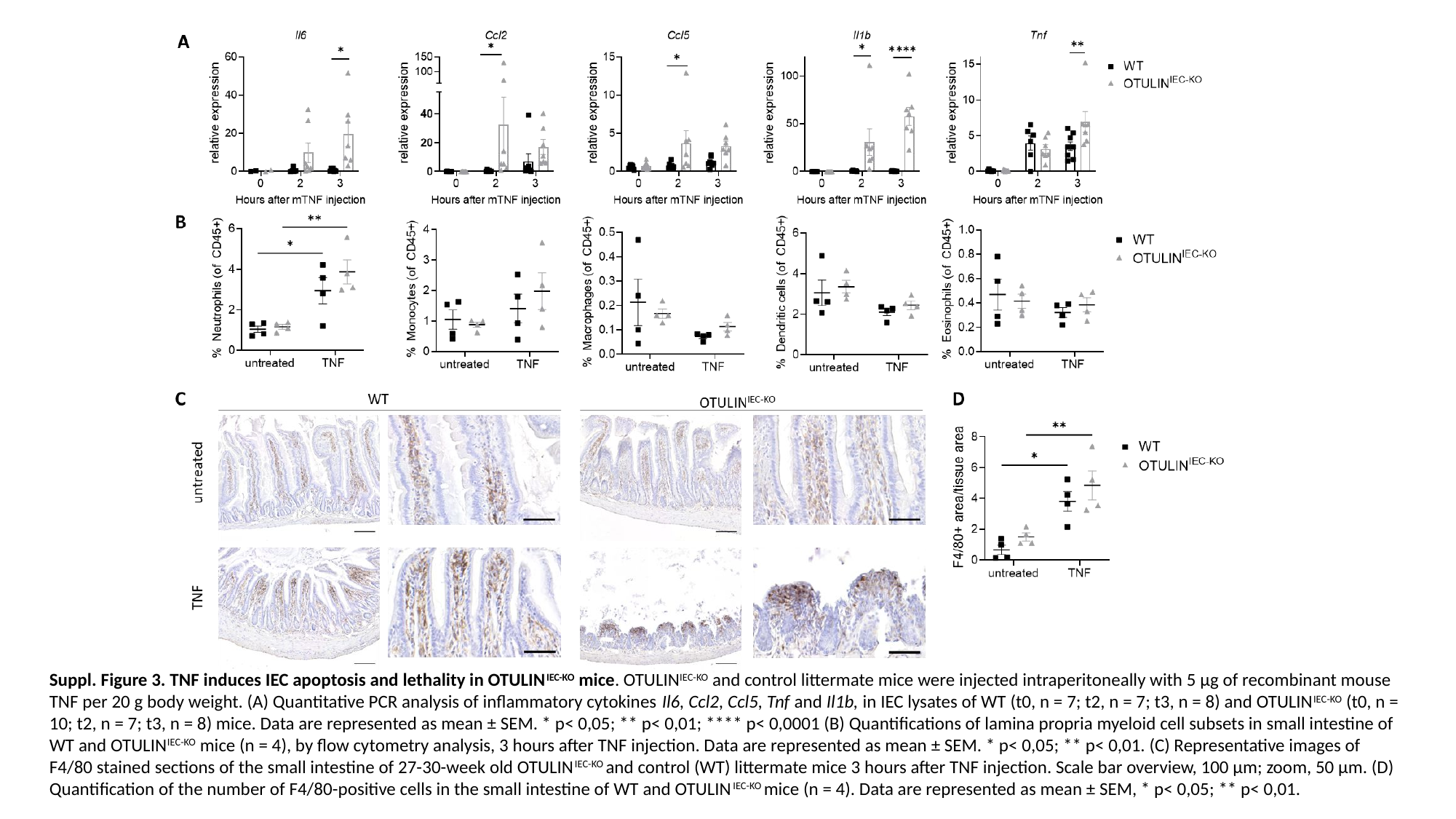

Suppl. Figure 3. TNF induces IEC apoptosis and lethality in OTULINIEC-KO mice. OTULINIEC-KO and control littermate mice were injected intraperitoneally with 5 µg of recombinant mouse TNF per 20 g body weight. (A) Quantitative PCR analysis of inflammatory cytokines Il6, Ccl2, Ccl5, Tnf and Il1b, in IEC lysates of WT (t0, n = 7; t2, n = 7; t3, n = 8) and OTULINIEC-KO (t0, n = 10; t2, n = 7; t3, n = 8) mice. Data are represented as mean ± SEM. * p< 0,05; ** p< 0,01; **** p< 0,0001 (B) Quantifications of lamina propria myeloid cell subsets in small intestine of WT and OTULINIEC-KO mice (n = 4), by flow cytometry analysis, 3 hours after TNF injection. Data are represented as mean ± SEM. * p< 0,05; ** p< 0,01. (C) Representative images of F4/80 stained sections of the small intestine of 27-30-week old OTULINIEC-KO and control (WT) littermate mice 3 hours after TNF injection. Scale bar overview, 100 μm; zoom, 50 μm. (D) Quantification of the number of F4/80-positive cells in the small intestine of WT and OTULINIEC-KO mice (n = 4). Data are represented as mean ± SEM, * p< 0,05; ** p< 0,01.

## Slide 4
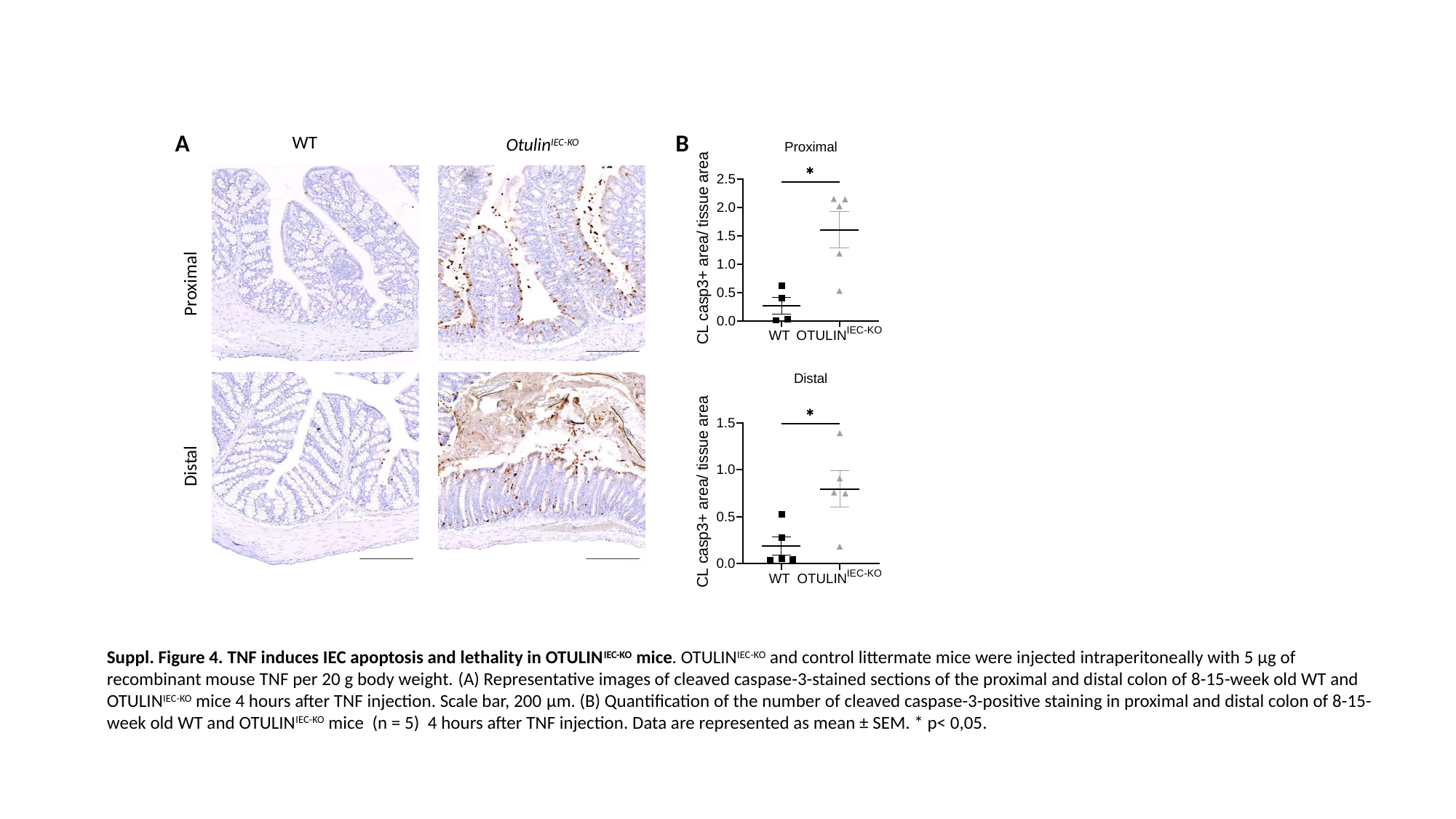

A
B
WT
OtulinIEC-KO
Proximal
Distal
Suppl. Figure 4. TNF induces IEC apoptosis and lethality in OTULINIEC-KO mice. OTULINIEC-KO and control littermate mice were injected intraperitoneally with 5 µg of recombinant mouse TNF per 20 g body weight. (A) Representative images of cleaved caspase-3-stained sections of the proximal and distal colon of 8-15-week old WT and OTULINIEC-KO mice 4 hours after TNF injection. Scale bar, 200 μm. (B) Quantification of the number of cleaved caspase-3-positive staining in proximal and distal colon of 8-15-week old WT and OTULINIEC-KO mice (n = 5) 4 hours after TNF injection. Data are represented as mean ± SEM. * p< 0,05.

## Slide 5
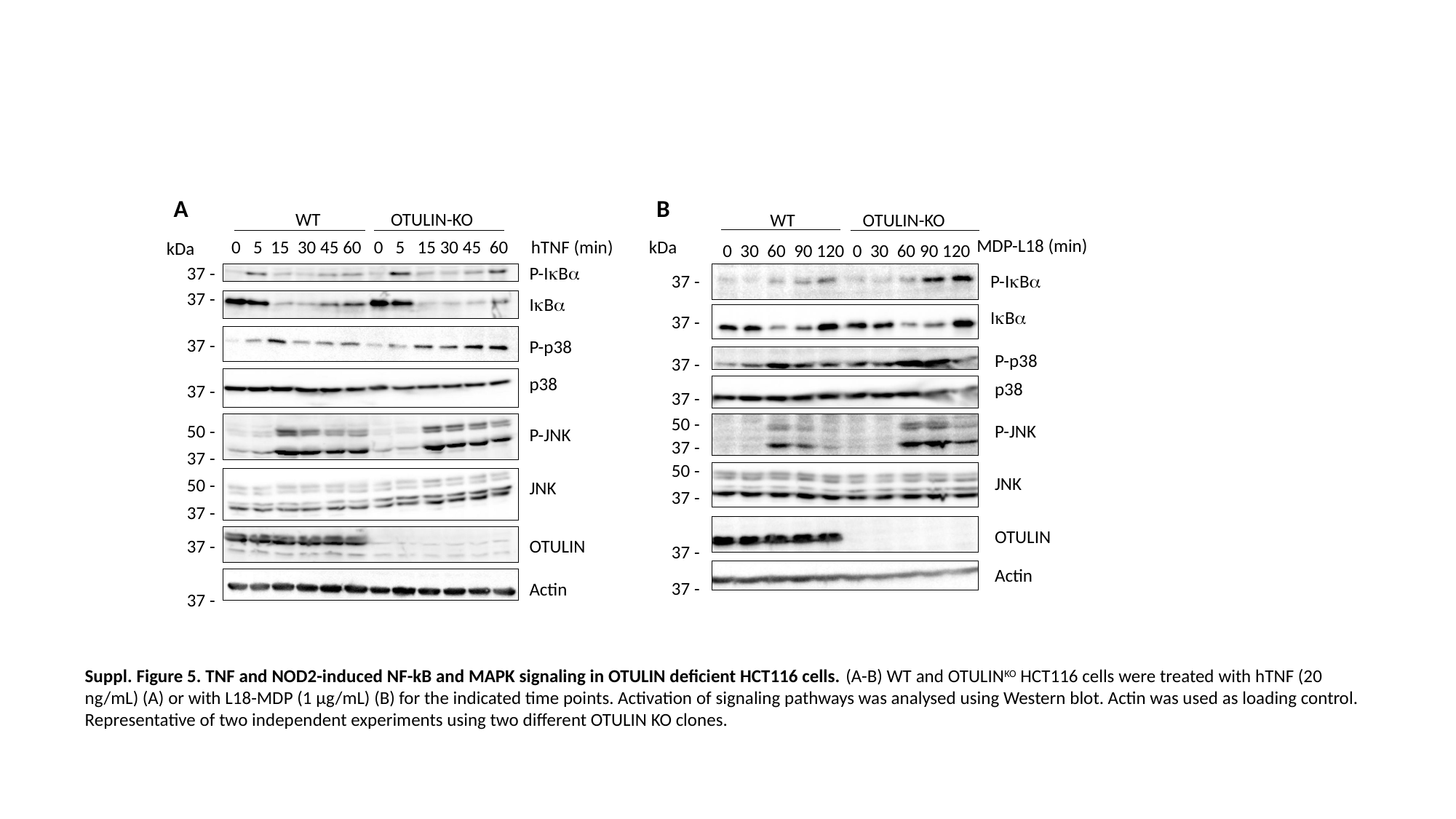

B
A
WT
OTULIN-KO
0 5 15 30 45 60 0 5 15 30 45 60
hTNF (min)
kDa
37 -
P-IkBa
37 -
IkBa
37 -
P-p38
p38
37 -
50 -
P-JNK
37 -
50 -
JNK
37 -
OTULIN
37 -
Actin
37 -
WT
OTULIN-KO
MDP-L18 (min)
kDa
0 30 60 90 120 0 30 60 90 120
37 -
37 -
P-p38
37 -
p38
37 -
50 -
P-JNK
37 -
50 -
JNK
37 -
OTULIN
37 -
Actin
37 -
P-IkBa
IkBa
Suppl. Figure 5. TNF and NOD2-induced NF-kB and MAPK signaling in OTULIN deficient HCT116 cells. (A-B) WT and OTULINKO HCT116 cells were treated with hTNF (20 ng/mL) (A) or with L18-MDP (1 µg/mL) (B) for the indicated time points. Activation of signaling pathways was analysed using Western blot. Actin was used as loading control. Representative of two independent experiments using two different OTULIN KO clones.

## Slide 6
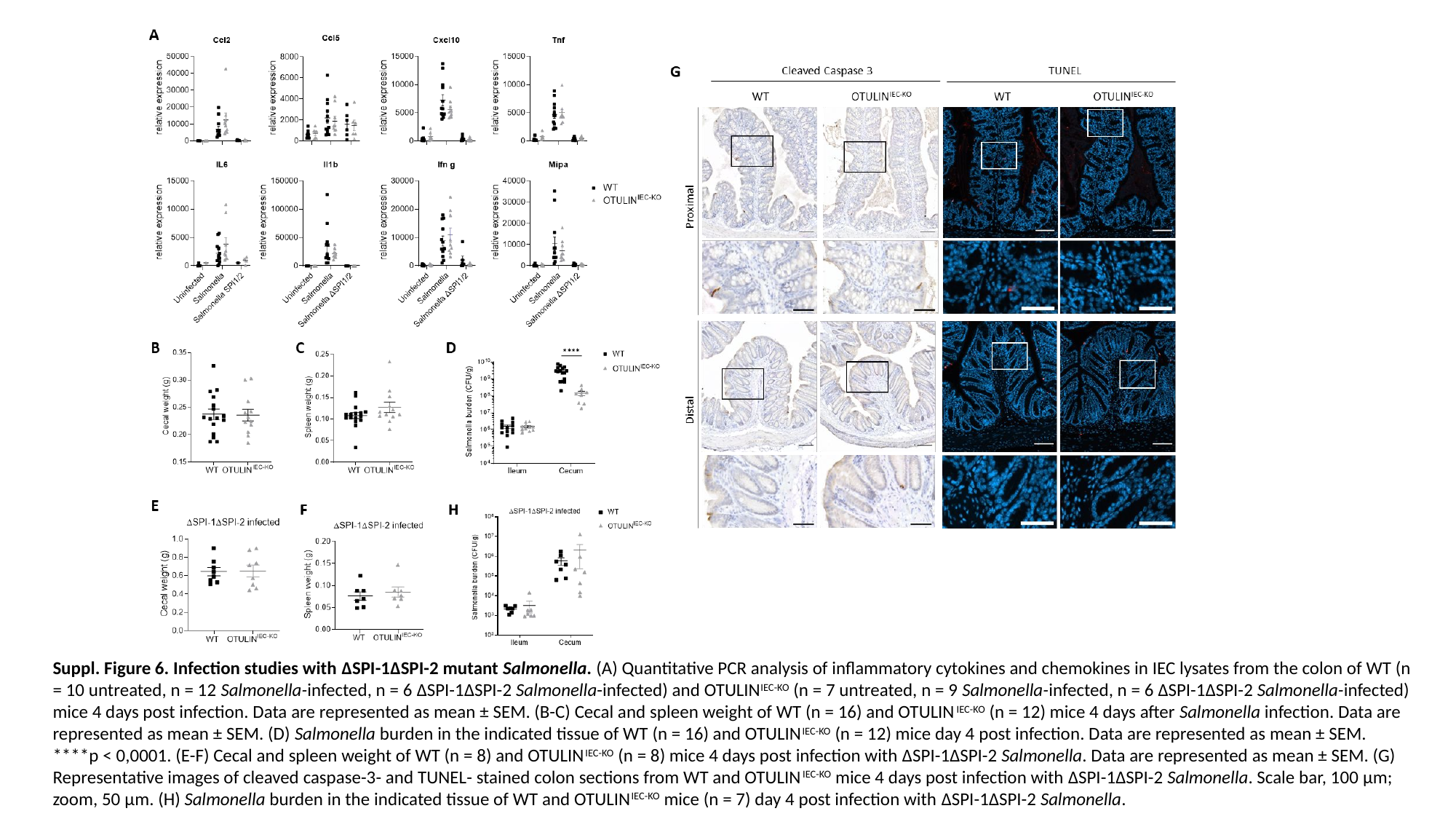

Suppl. Figure 6. Infection studies with ΔSPI-1ΔSPI-2 mutant Salmonella. (A) Quantitative PCR analysis of inflammatory cytokines and chemokines in IEC lysates from the colon of WT (n = 10 untreated, n = 12 Salmonella-infected, n = 6 ΔSPI-1ΔSPI-2 Salmonella-infected) and OTULINIEC-KO (n = 7 untreated, n = 9 Salmonella-infected, n = 6 ΔSPI-1ΔSPI-2 Salmonella-infected) mice 4 days post infection. Data are represented as mean ± SEM. (B-C) Cecal and spleen weight of WT (n = 16) and OTULINIEC-KO (n = 12) mice 4 days after Salmonella infection. Data are represented as mean ± SEM. (D) Salmonella burden in the indicated tissue of WT (n = 16) and OTULINIEC-KO (n = 12) mice day 4 post infection. Data are represented as mean ± SEM. ****p < 0,0001. (E-F) Cecal and spleen weight of WT (n = 8) and OTULINIEC-KO (n = 8) mice 4 days post infection with ΔSPI-1ΔSPI-2 Salmonella. Data are represented as mean ± SEM. (G) Representative images of cleaved caspase-3- and TUNEL- stained colon sections from WT and OTULINIEC-KO mice 4 days post infection with ΔSPI-1ΔSPI-2 Salmonella. Scale bar, 100 μm; zoom, 50 μm. (H) Salmonella burden in the indicated tissue of WT and OTULINIEC-KO mice (n = 7) day 4 post infection with ΔSPI-1ΔSPI-2 Salmonella.
