# Supplementary material for: OTULIN protects the intestinal epithelium from apoptosis during inflammation and infection
Source: Cell Death Dis. 2023 Aug 19;14(8):534. doi: 10.1038/s41419-023-06058-7 (PMC10439912; doi:10.1038/s41419-023-06058-7)

FIGURE 1B

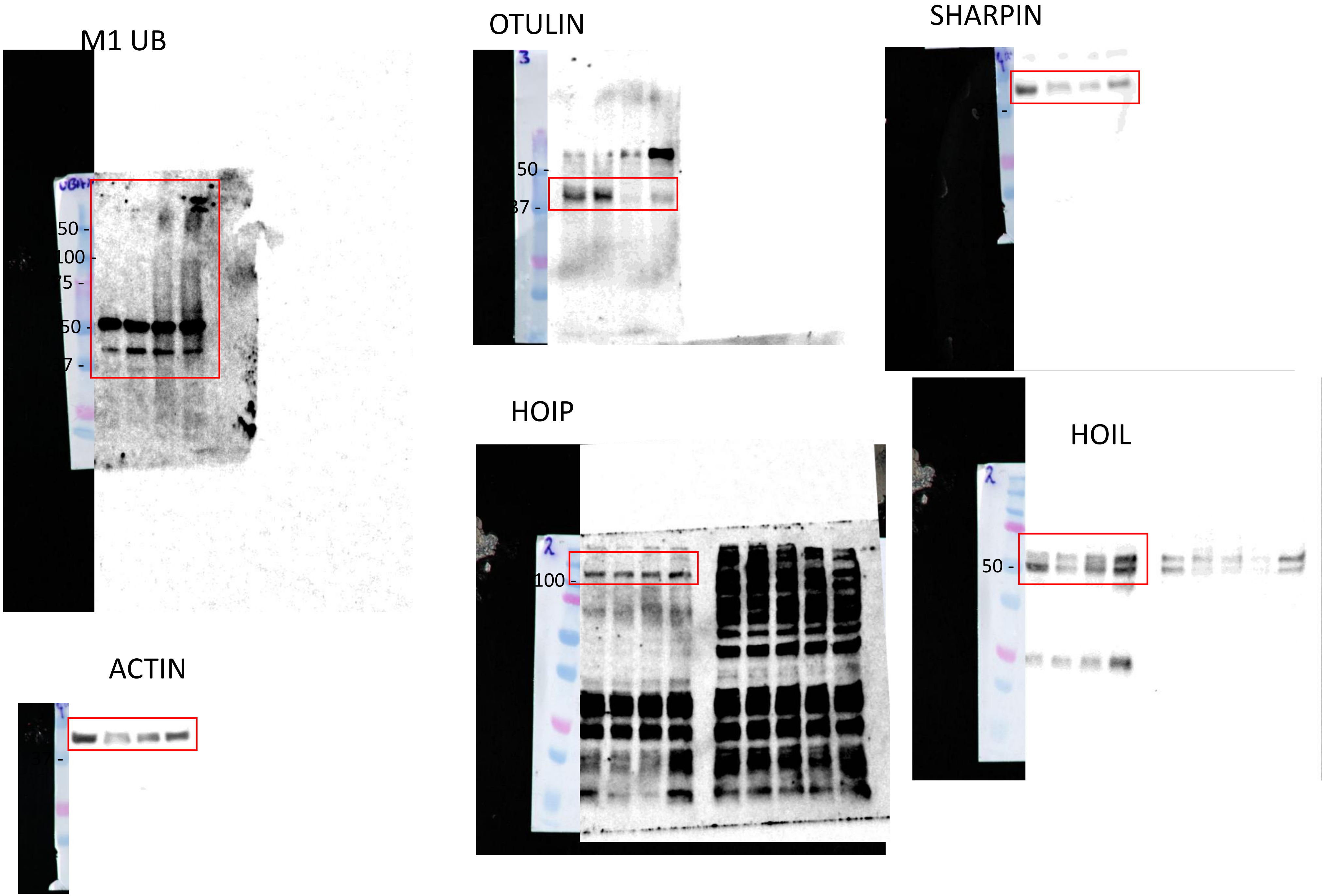

FIGURE 1C

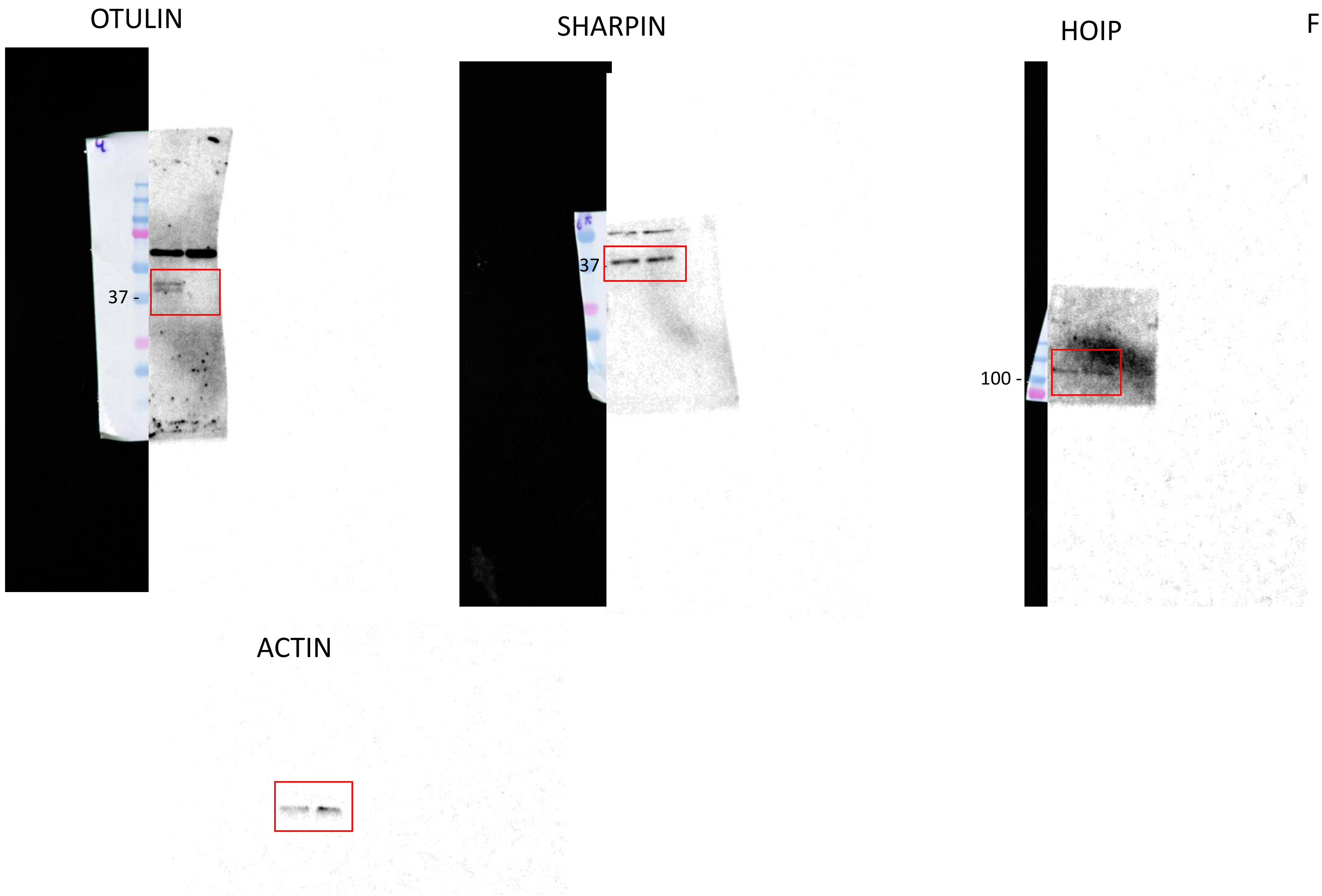

FIGURE 4C

Caspase 3

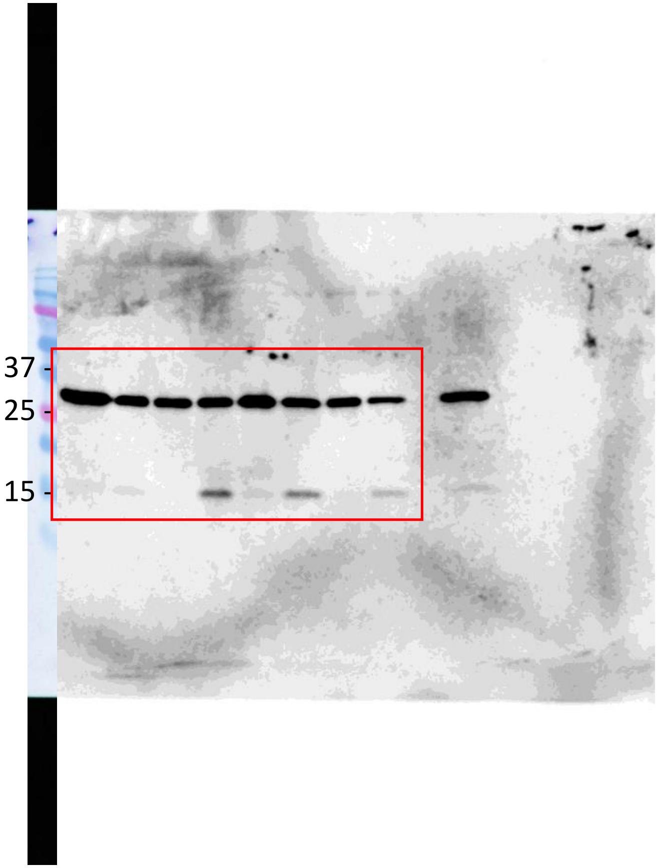

ACTIN

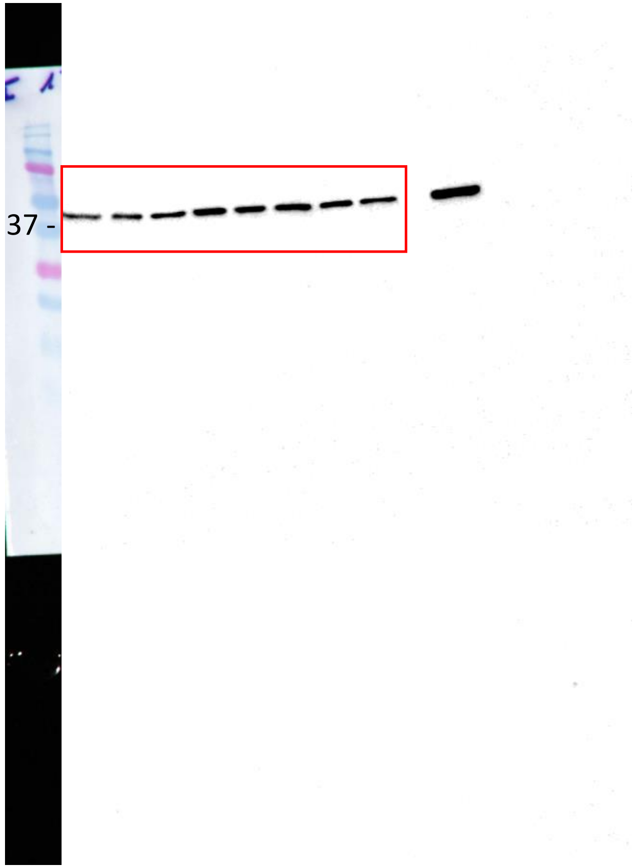

FIGURE 4E

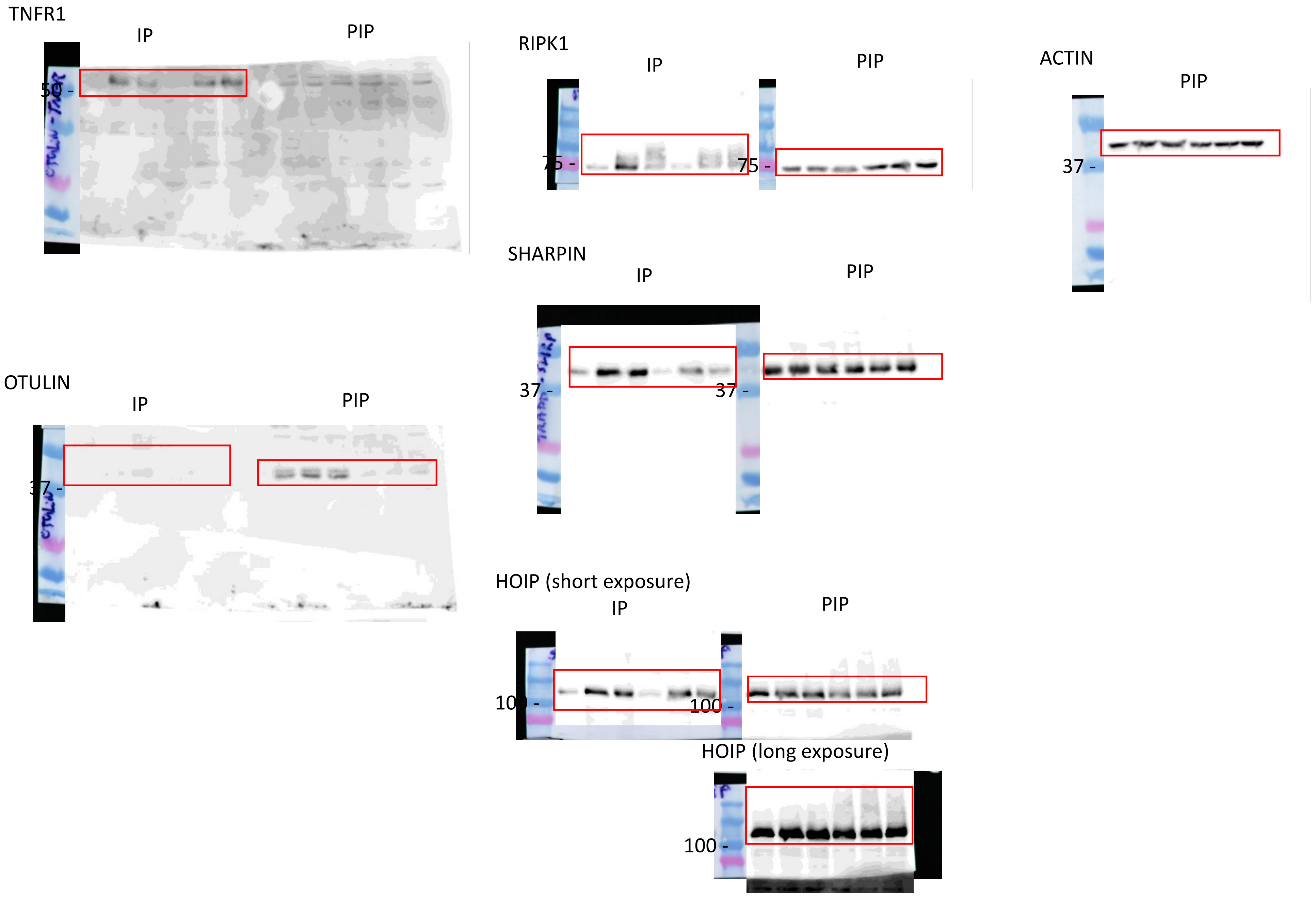
TNFR1
IP
PIP
50 -
RIPK1
IP
PIP
75 -
75 -
ACTIN
PIP
37 -
SHARPIN
IP
PIP
37 -
37 -
OTULIN
IP
PIP
37 -
HOIP (short exposure)
IP
PIP
100 -
100 -
HOIP (long exposure)
100 -

FIGURE 4F

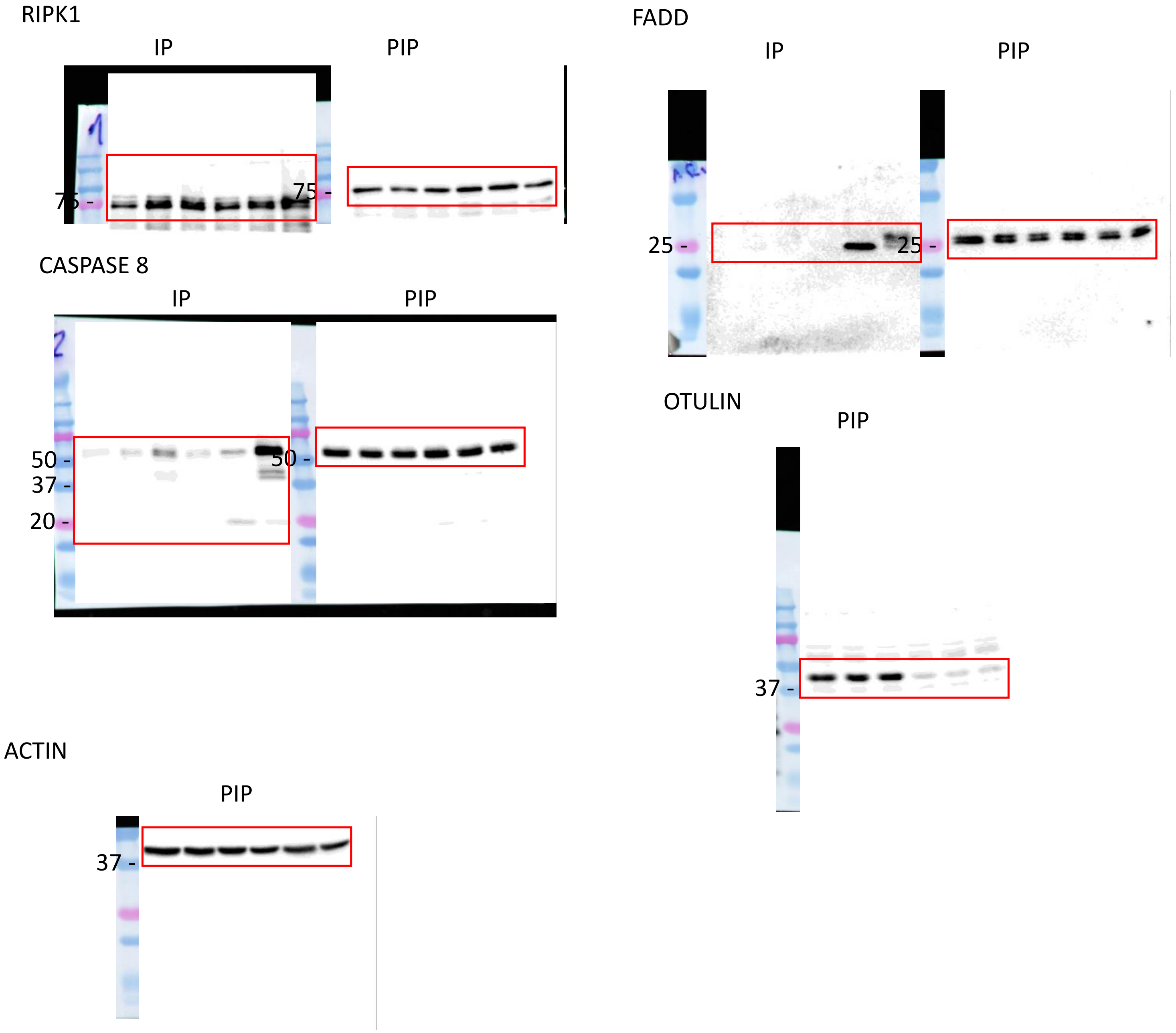
RIPK1
IP
PIP
75 -
75 -
FADD
IP
PIP
25 -
25 -
CASPASE 8
IP
PIP
50 -
37 -
20 -
50 -
OTULIN
PIP
37 -
ACTIN
PIP
37 -

Suppl. FIGURE 5A

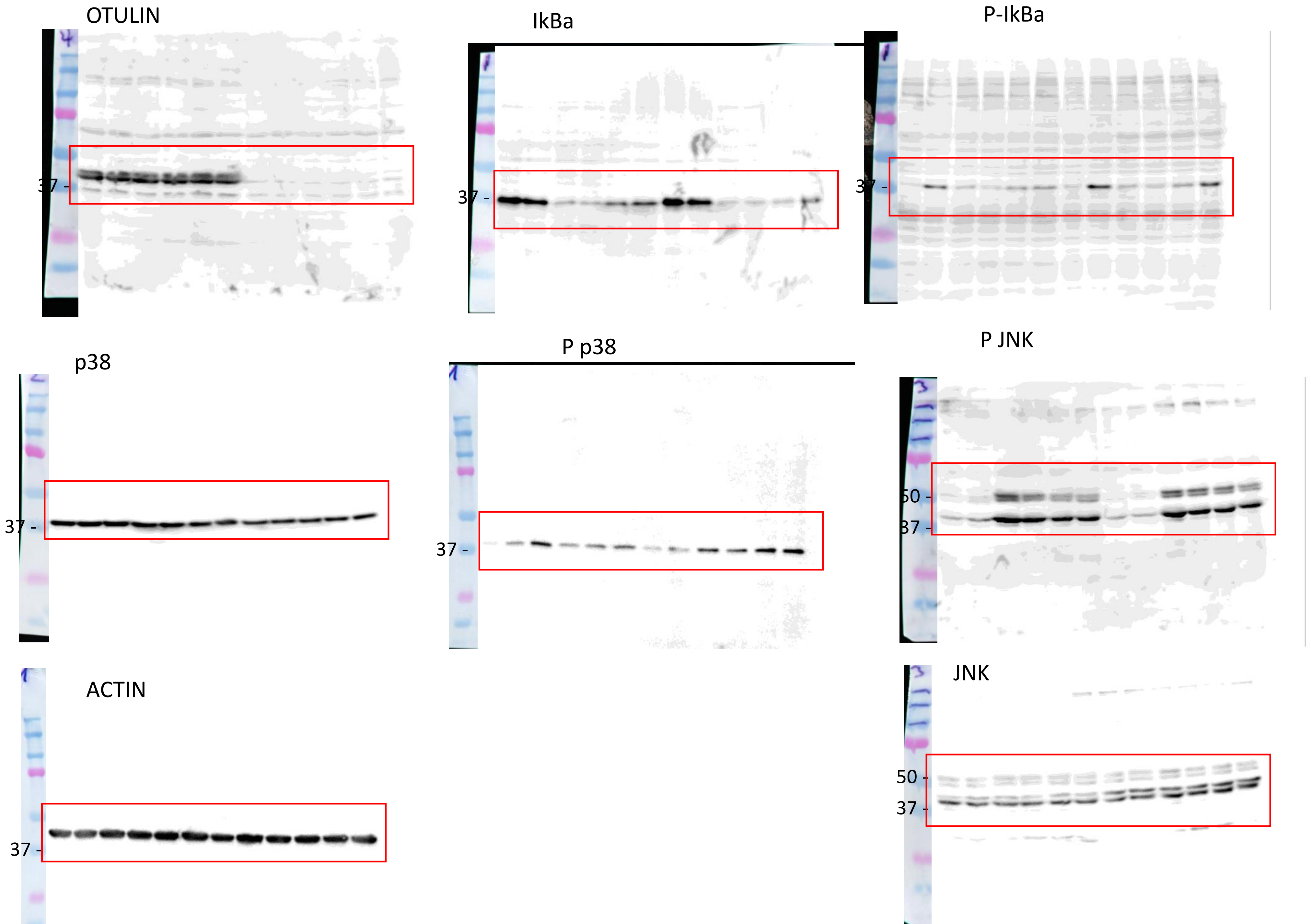

Suppl. FIGURE 5B

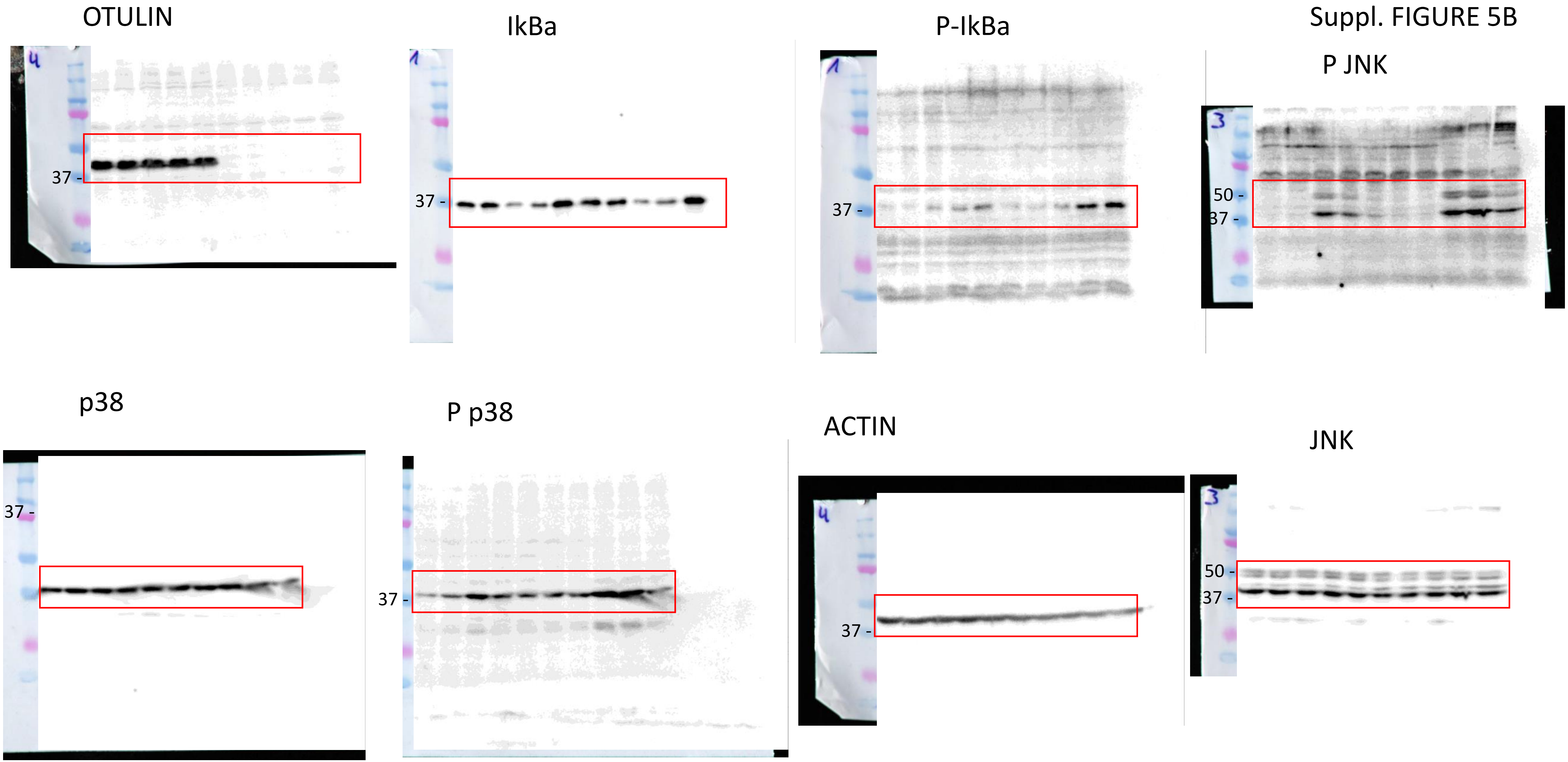

Supplement: Supplementary file 2 — uncropped western blot file [file 41419_2023_6058_MOESM2_ESM.pdf]
